# Redox signaling by glutathione peroxidase 2 links vascular modulation to metabolic plasticity of breast cancer

**DOI:** 10.1073/pnas.2107266119

**Published:** 2022-02-22

**Authors:** Zuen Ren, Huizhi Liang, Phillip M. Galbo, Malindrie Dharmaratne, Ameya S. Kulkarni, Atefeh Taherian Fard, Marie Louise Aoun, Nuria Martinez-Lopez, Kimita Suyama, Outhiriaradjou Benard, Wei Zheng, Yang Liu, Joseph Albanese, Deyou Zheng, Jessica C. Mar, Rajat Singh, Michael B. Prystowsky, Larry Norton, Rachel B. Hazan

**Affiliations:** ^a^Department of Pathology, Albert Einstein College of Medicine, Bronx, NY 10461;; ^b^Department of Genetics, Albert Einstein College of Medicine, Bronx, NY 10461;; ^c^Department of Microbiology and Immunology, Albert Einstein College of Medicine, Bronx, NY 10461;; ^d^Australian Institute for Bioengineering and Nanotechnology, University of Queensland, Brisbane, 4072 QLD, Australia;; ^e^Department of Endocrinology, Albert Einstein College of Medicine, Bronx, NY 10461;; ^f^Department of Hematology and Medical Oncology, The Tisch Cancer Institute, Icahn School of Medicine at Mount Sinai, New York, NY 10029;; ^g^Department of Neurology, Albert Einstein College of Medicine, Bronx, NY 10461;; ^h^Department of Medicine, Memorial Sloan-Kettering Cancer Center, New York, NY 10021

**Keywords:** breast cancer, glutathione peroxidase 2, ROS signaling, HIF1α, metabolism

## Abstract

In search of redox mechanisms in breast cancer, we uncovered a striking role for glutathione peroxidase 2 (GPx2) in oncogenic signaling and patient survival. GPx2 loss stimulates malignant progression due to reactive oxygen species/hypoxia inducible factor-α (HIF1α)/VEGFA (vascular endothelial growth factor A) signaling, causing poor perfusion and hypoxia, which were reversed by GPx2 reexpression or HIF1α inhibition. Ingenuity Pathway Analysis revealed a link between GPx2 loss, tumor angiogenesis, metabolic modulation, and HIF1α signaling. Single-cell RNA analysis and bioenergetic profiling revealed that GPx2 loss stimulated the Warburg effect in most tumor cell subpopulations, except for one cluster, which was capable of oxidative phosphorylation and glycolysis, as confirmed by coexpression of phosphorylated-AMPK and GLUT1. These findings underscore a unique role for redox signaling by GPx2 dysregulation in breast cancer, underlying tumor heterogeneity, leading to metabolic plasticity and malignant progression.

## Significance

Redox regulation of breast cancer underlies malignant progression. Loss of the antioxidant glutathione peroxidase 2 in breast cancer cells increases reactive oxygen species, thereby activating hypoxia inducible factor-α (HIF1α) signaling. This in turn causes vascular malfunction, resulting in hypoxia and metabolic heterogeneity. HIF1α suppresses oxidative phosphorylation and stimulates glycolysis (the Warburg effect) in most of the tumor, except for one cancer subpopulation, which was capable of using both metabolic modalities. Hence, adopting a hybrid metabolic state may allow tumor cells to survive under aerobic or hypoxic conditions, a vulnerability that may be exploited for therapeutic targeting by either metabolic or redox-based strategies.

Tumor cell hyperproliferation results in cell crowding, causing nutrients and oxygen deprivation, leading to hypoxia (1). To meet the energetic demands of cancer cells, mitochondria consume the cellular oxygen, resulting in oxidative phosphorylation, leading to reactive oxygen species (ROS) production (2).

While low to mild ROS levels promote oncogenic signaling and malignancy, high levels of ROS cause DNA damage and apoptosis (3, 4), an effect that is often coopted by chemotherapy or radiation to target cancer cells (5). Tumor cells evade ROS cytotoxicity by increasing the expression of antioxidant enzymes, such as superoxide dismutase, periodoxin-theriodoxin, catalases, and glutathione peroxidases (6, 7), which generally convert hydrogen peroxide produced by mitochondrial electron leak into water using glutathione (8).

ROS are known to stimulate oncogenic signaling with special emphasis on hypoxia inducible factor-α (HIF1α). ROS stabilize HIF1α protein via inhibition of the oxygen sensing propyl hydroxylase protein D (PHD), which normally mark HIF1α for proteasomal degradation (9, 10). HIF1α promotes malignancy via effects on tumor angiogenesis, proliferation, epithelial to mesenchymal transition (EMT), stemness, and glucose metabolism (1, 11). HIF1α stimulates vascular endothelial growth factor A (VEGFA) gene transcription, which promotes angiogenesis, thereby increasing nutrient availability and oxygen supply to hypoxic tumor areas (12, 13). Paradoxically, VEGFA overproduction may also cause vascular malfunction, resulting in immature or poorly perfusing vessels, thereby exacerbating hypoxia (14). This further stabilizes HIF1α protein, which shifts cells from oxidative phosphorylation (OXPHOS) to aerobic glycolysis, known as the Warburg effect (12, 15). While OXPHOS generates high levels of ATP as compared to glycolysis, tumor cells leverage glucose metabolism to generate building blocks for biomass biosynthesis (16). However, aggressive cancer cells were also shown to be able to use OXPHOS and glycolysis, which might be necessary to survive under hypoxic and aerobic conditions that can be encountered at the primary tumor, in circulation, or at metastatic sites (17, 18).

A comparison of carcinoma cell lines derived from the polyoma middle T (PyMT) mammary tumor model unraveled a dramatic down-regulation of glutathione peroxidase 2 (GPx2) in metastatic relative to nonmetastatic cells from the parental tumor. Moreover, the loss of GPx2 in several molecular breast cancer (BC) subtypes was correlated with poor patient survival, underscoring the clinical significance of GPx2 loss in BC. GPx2 knockdown (KD) in murine and human BC cells stimulates ROS/HIF1α/VEGFA signaling, which enhanced malignant progression via vascular modulation, resulting in poor perfusion, hypoxia, and a shift from OXPHOS to aerobic glycolysis (the Warburg effect). Transcriptomic analysis of single-cell RNA-sequencing (scRNA-seq) data and bioenergetic profiling confirmed that GPx2 KD stimulated the Warburg effect in most clusters, except for cluster 5, which was able of using OXPHOS and glycolysis. The latter was confirmed by coexpression of phosphorylated AMPK and Glut1 in discrete tumor areas, which was markedly increased in the PyMT1/GPx2 KD tumor. These findings underscore the profound effects of GPx2 loss on redox signaling, which in turn drives tumor heterogeneity, causing metabolic plasticity and malignant progression.

## Results

### GPx2 Down-Regulation in Mammary Tumor Cells Is Associated with Metastatic Potential.

Oxidative stress is a hallmark of malignant tumors due to ROS accumulation, which causes damage to lipids, protein, and DNA, resulting in activation of redox signaling that lowers ROS below lethal threshold (19). Using carcinoma cell lines derived from the PyMT mammary tumor model (20), we found a dramatic down-regulation of the antioxidant GPx2 in the highly metastatic PyMT2 relative to the weakly metastatic PyMT1 tumor cell line, which were representative of other cell lines derived from the same mammary tumor. PyMT2 cells generated a high volume of lung metastatic nodules (number and diameter) relative to PyMT1 cells following mammary fat pad injection (*SI Appendix*, Fig. S1 *A*–*C*), and were interestingly devoid of GPx2 expression relative to PyMT1 cells (*SI Appendix*, Fig. S1*D*). Consistent with GPx2 antioxidant activity, PyMT1 cells produced lower levels of ROS than PyMT2 cells (*SI Appendix*, Fig. S1*E*). These data suggested a relationship between GPx2 loss, ROS, and metastasis. To confirm whether GPx2 regulates malignant progression, we knocked down GPx2 in PyMT1 cells using short-hairpin RNAs (shRNAs). Three of five shRNA hairpins were efficient in silencing GPx2 expression relative to a control nontargeting hairpin sequence (*SI Appendix*, Fig. S1 *F*, *Upper*). As expected, GPx2 KD markedly increased ROS production in cells that were untreated or treated with H_2_O_2_ to enhance oxidation (*SI Appendix*, Fig. S1 *F*, *Lower*). Moreover, GPx2 loss caused increases in cell growth in two-dimensional and three-dimensional cultures, as well as in invasion through Matrigel *SI Appendix*, Fig. S1 *G*–*I*). Thus, these data suggested a link between GPx2 loss, ROS, and malignant transformation.

### GPx2 Loss in Human Breast Cancer Is Associated with Oncogenic Signaling and Poor Patient Survival.

To evaluate the clinical relevance of GPx2 loss in human BC, we examined the relationship between GPx2 expression, oncopathway activation, and patient survival. Analysis of GPx2 mRNA expression from The Cancer Genome Atlas (TCGA) BC datasets (BRCA) revealed that GPx2 was significantly attenuated in the tumor relative to matched normal breast tissue (*SI Appendix*, Fig. S2*A*). Further examination of BC subtypes showed that GPx2 mRNA was down-regulated in HER2-enriched relative to luminal A and B tumors, as well as in Basal-like (the great majority of triple-negative BC, TNBC) tumors relative to all other subtypes (*SI Appendix*, Fig. S2*B*), implying a link between GPx2 loss and pathological progression. Indeed, Kaplan–Meier analysis of the Gene Expression Omnibus BC dataset, containing gene-expression profiles from 1,809 BC patients (21), showed an association between low GPx2 mRNA in luminal B, HER2-enriched, Basal-like tumors, and poor patient survival (*SI Appendix*, Fig. S2*C*). Consistent with these findings, gene set enrichment analysis (GSEA) revealed that, except for luminal A, BCs with low GPx2 expression showed enrichment in oncogenic pathways compared to high-GPx2 expressing tumors (*SI Appendix*, Fig. S2 *D*–*J*). Low GPx2-expressing luminal B and HER2-amplified tumors were enriched in estrogen-regulated cell growth (early and late estrogen response), hypoxia, Notch, and NF-κB signaling pathways (*SI Appendix*, Fig. S2 *E*–*H*). By comparison, HER2-enriched and Basal-like cancers were both enriched in proliferation or cell cycle pathways (mitotic spindle, G2M checkpoint, E2F target genes) (*SI Appendix*, Fig. S2 *G*–*J*). Interestingly, all three subtypes showed effects of GPx2 loss on EMT signaling, including Notch and TGF-β (*SI Appendix*, Fig. S2 *E*, *G*, and *I*). Finally, glycolysis was mostly enriched in Basal-like tumors, and to a less significant extent, also in luminal B tumors, which could be due to high proliferative index of these cancer types (*SI Appendix*, Fig. S2 *I* and *E*). In sum, GPx2 loss exert broad effects on the tumor phenotype that are consistent with ROS oncogenic signaling.

**Fig. 1.**
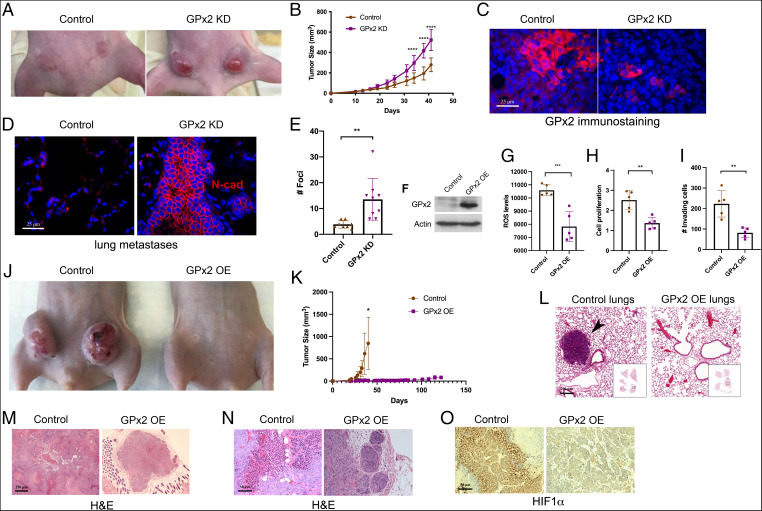
Opposing effects of GPx2 loss- and gain-of-function on mammary tumor growth and metastasis. (*A*) Control PyMT1 and PyMT1/GPx2 KD (sh3) cell lines (1 × 10^6^) were bilaterally injected into mammary fat pads of female athymic nude mice (*n* = 5). A representative mouse of each group is shown. (*B*) Tumor growth over 42 d posttumor onset is shown as tumor volume; mean ± SEM; ****P < 0.0001. (*C*) Control and GPx2 KD tumors (*n* = 10; 2 tumors per mouse) were immunostained for GPx2. Representative images are shown. (*D*) End point spontaneous lung metastasis was assessed by counting tumor foci in H&E-stained lungs and confirmed by *N*-cadherin immunostaining. (*E*) The number and distribution of lung foci generated by PyMT1/GPx2 KD versus PyMT1 control cell lines is shown as mean ± SEM; **P < 0.01. (*F*) PyMT2 cells were transduced with control vector or mouse GPx2 lentiviral vector. Cells were immunoblotted for GPx2 and actin, or (*G*) tested for ROS levels in control PyMT2 and PyMT2/GPx2 OE cells using H2DCFDA fluorescence (mean ± SEM; ***P < 0.001), or (*H*) tested for cell proliferation at 24 h postplating using the WST-1 assay readout (450 nm) (mean ± SEM; **P < 0.01), or (*I*) assayed for invasion in Matrigel-coated transwells (mean ± SEM; **P < 0.01). (*J*) PyMT2 control and GPx2 OE cells were bilaterally injected into mammary fat pads of female athymic nude mice (PyMT2; *n* = 4) and GPx2 OE (*n* = 5); representative images of tumor growth at 40 d postonset are shown. (*K*) Tumor growth curves over 40 d after tumor onset (control compared to GPx2 OE mice versus 125 d GPx2 OE mice only) are shown as mean ± SEM; *P < 0.05. (*L*) H&E-stained lung sections from scans of whole lung lobes (boxes) from mice carrying control or GPx2 OE tumors. (*M* and *N*) H&E-stained images (4× and 20× magnification) of control and GPx2 OE tumors are shown. (*O*) Representative HIF1α immunohistochemistry of control and GPx2 OE tumors (*n* = 8; 2 tumors per mouse).

In contrast to GPx2, the expression of GPx1 or GPx3 mRNA expression in all BC subtypes was not correlated with patient survival duration (*P* > 0.05) (*SI Appendix*, Fig. S3 *A* and *B*). By comparison, high GPx4 expression in Basal-like BCs was significantly associated with worse survival (GPx4, hazard ratio = 1.4 [1.09–1.8], *P* = 0.0089) (*SI Appendix*, Fig. S3*C*), which agrees with effects on EMT and chemoresistance (22). Finally, due to undetected levels of GPx5 or GPx6 mRNA in the TCGA BC datasets, the association of these GPxs with patient survival was not assessed (*SI Appendix*, Fig. S3 *D* and *E*). Hence, of all the GPx family, GPx2 appears to be the most clinically relevant for elucidating the effects of redox signaling on BC progression.

### GPx2 KD in PyMT Tumor Cells Enhances Primary Tumor Growth and Metastasis.

To determine whether GPx2 regulates malignant progression in vivo, we tested whether GPx2 KD in mouse and human BC cells affects tumor growth or metastasis. Mammary fat pad injection of control PyMT1 and PyMT1/GPx2 KD (sh3) cells into female athymic nude mice resulted in the formation of substantially large tumors by PyMT1/GPx2 KD cells as compared to PyMT1 control cells, especially at 20 to 45 d posttumor onset (Fig. 1 *A* and *B*). Of note, GPx2 KD tumors were notoriously reddish, implying angiogenesis (Fig. 1*A*). GPx2 immunostaining confirmed reduced GPx2 levels in KD relative to control tumors (Fig. 1C). Moreover, GPx2 KD tumors were dramatically metastatic relative to control PyMT1 tumors, as noted by *N*-cadherin staining of lung foci (Fig. 1 *D*–*E*). These findings demonstrate that GPx2 loss accelerates tumor growth and metastasis.

**Fig. 2.**
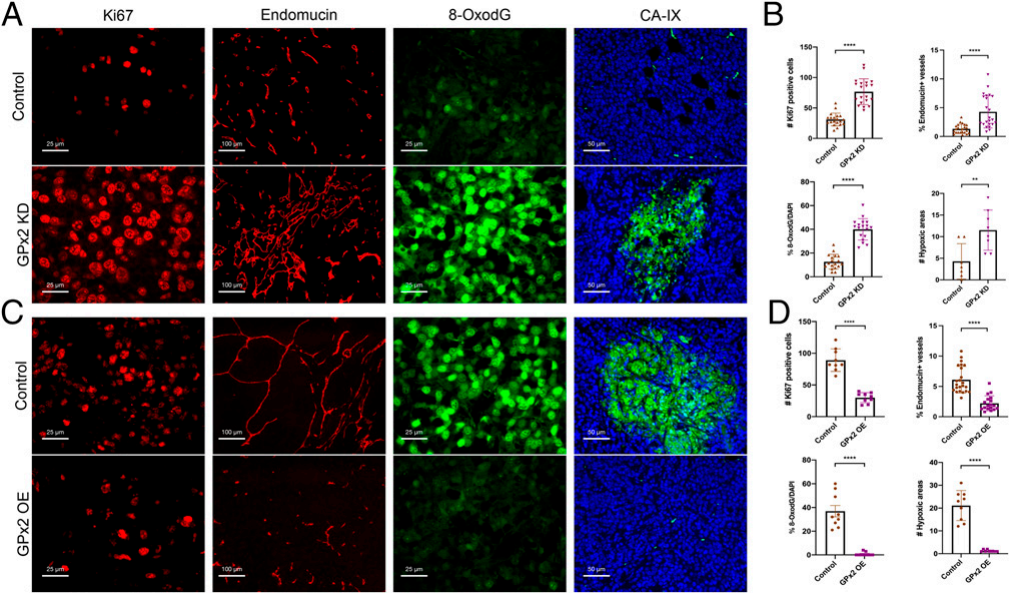
GPx2 loss results in tumor cell proliferation, abnormal angiogenesis, oxidative stress, and intratumor hypoxia. (*A* and *B*) PyMT1/GPx2 KD (sh3) versus PyMT1 control mammary tumors from five mice (two tumors per mouse) or (*C* and *D*) GPx2 OE PyMT2 tumors (GPx2OE) versus PyMT2 control tumors from four mice (two tumors per mouse) were immunostained with indicated antibodies (two random sections each). Per section, (*A*–*D*) the number of *K*_i_67^+^ tumor cells, mean ± SEM; the fraction of endomucin-labeled vessels was quantified by ImageJ, mean ± SEM; the percentage of 8-OxodG^+^ cells, mean ± SEM; the number of CA-IX^+^ (hypoxic) areas, mean ± SEM; ****P < 0.0001; **P < 0.01.

### Exogenous GPx2 Expression in Metastatic PyMT2 Cells Suppresses Tumor Growth and Metastasis.

We next expressed GPx2 in the highly metastatic PyMT2 cells, which are devoid of endogenous GPx2 expression, to determine effects on malignant growth and metastatic seeding. GPx2 expression in PyMT2 cells caused significant reductions in ROS production, cell growth, and Matrigel invasion (Fig. 1 F–I). Moreover, GPx2 caused dramatic suppression of mammary tumor growth relative to control tumors (Fig. 1 J and K). While control tumors reached a maximal volume by 30 d postinjection, GPx2 expressing PyMT2 cells grew into minimal lumps even after 100 d of incubation (Fig. 1K), which were in turn nonmetastatic relative to PyMT2 controls (Fig. 1L). Clearly, GPx2 overexpressing (OE) PyMT2 tumors were glandular relative to the de-differentiated control PyMT2 tumors (Fig. 1 M and N), as well as nonhypoxic, as indicated by HIF1α immunostaining (Fig. 1O). Together, these findings underscore a robust suppressive effect of GPx2 on tumor progression.

### GPx2 KD Increases Cell Proliferation, Angiogenesis, Oxidative Stress, and Hypoxia In Vivo.

To elucidate the biological processes regulated by GPx2, we examined in vivo the effect of GPx2 KD on tumor cell proliferation, angiogenesis, oxidative stress, and hypoxia, all hallmarks of malignancy. *K*_i_67 immunostaining revealed that PyMT1/GPx2 KD tumors were highly proliferative (Fig. 2*A*, first panels, and Fig. 2*B*), whereas staining for endomucin, an endothelial membrane glycoprotein (23), showed a fourfold increase in vessel density (Fig. 2*A*, second panels, and Fig. 2*B*). Of note, vessels in GPx2 KD tumors were convoluted and tortuous, implying aberrant angiogenesis (Fig. 2*A*, second panels). The effect of GPx2 loss on oxidative stress was measured by the production of 8-hydroxy-2-deoxyguanosine (8-OxodG), a marker of ROS-induced DNA damage (24). 8-OxodG staining was markedly increased in GPx2 KD relative to control tumors (Fig. 2*A*, third panels, and Fig. 2*B*). Consistent with effects of ROS on hypoxia, staining of tumors for carbonic anhydrase 9 (CA-IX), a hypoxia marker (25), showed a notable increase in CA-IX^+^ areas in GPx2 KD tumors (Fig. 2*A*, fourth panels, and Fig. 2*B*). Interestingly, all these GPx2 KD stimulated events were reversed by exogenous expression of GPx2 in highly metastatic PyMT2 cells. This was shown by dramatically reduced tumor cell proliferation (*K*_i_67) (Fig. 2*C*, first panels, and Fig. 2*D*), angiogenesis (endomucin) (Fig. 2*C*, second panels, and Fig. 2*D*), ROS-induced DNA damage (8-OxodG) (Fig. 2*C*, third panels, and Fig. 2*D*), and hypoxia (CA-IX) (Fig. 2*C*, fourth panels, and Fig. 2*D*). Hence, GPx2 loss activates key biological events underlying malignancy, likely due to ROS signaling.

**Fig. 3.**
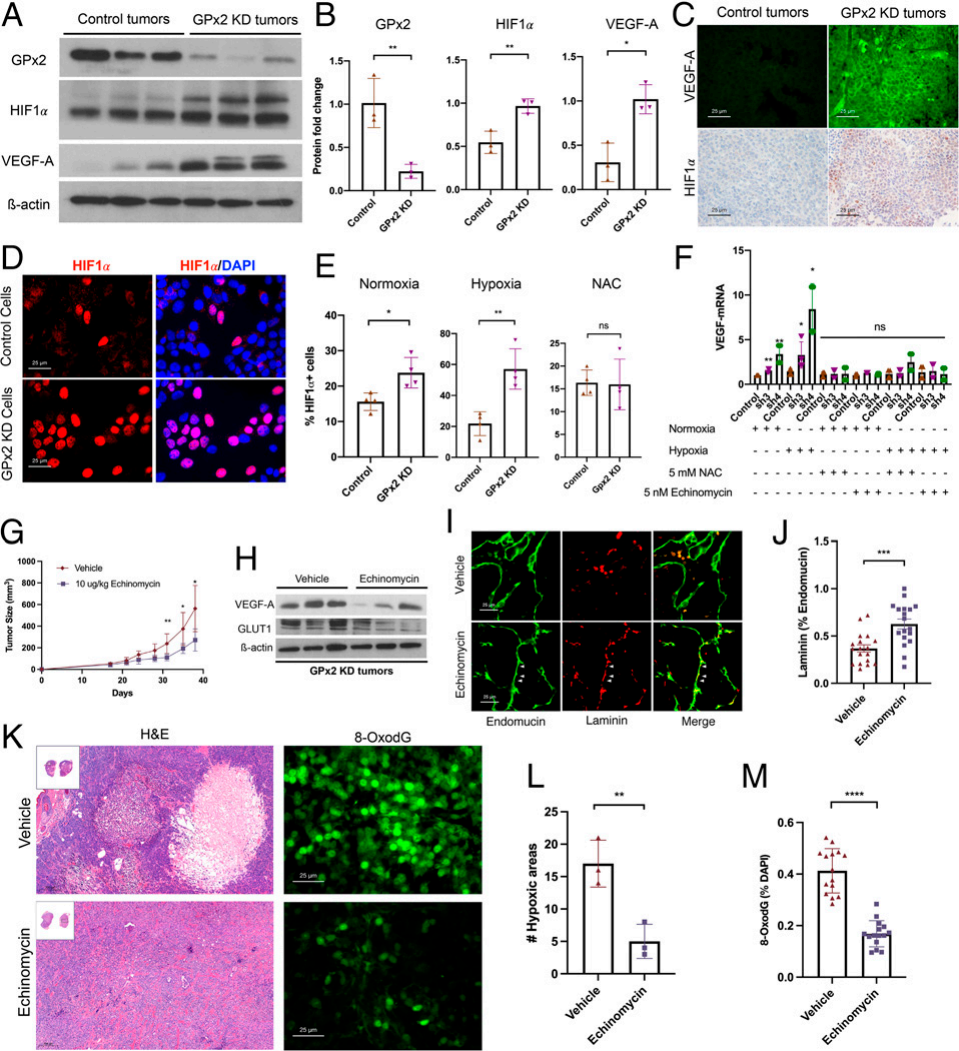
GPx2 KD stimulates ROS/HIF1α/VEGFA signaling leading to angiogenesis and tumor growth that is attenuated by HIF1α inhibition by echinomycin. (*A*) PyMT1 control and PyMT1/GPx2 KD tumor lysates (*n* = 3 from 3 independent mice) were immunoblotted with anti GPx2, VEGFA, HIF1α, or actin antibody. (*B*) Quantification of immunoblots of GPx2, HIF1α, VEGFA protein levels relative to actin in PyMT1/GPx2 KD relative to PyMT1 control tumors are displayed as bar graphs; mean ± SEM; *P < 0.05, **P < 0.01. (*C*) PyMT1 control and PyMT1/GPx2 KD tumors were immunostained with anti VEGFA (FITC) or HIF1α (DAB) antibody. (*D*) PyMT1 control and PyMT1/GPx2 KD cells were cultured under normoxia (20% oxygen) or hypoxia (1% oxygen) for 24 h ± 5 mM NAC, and immunostained with anti-HIF1α; representative images of cells stained under hypoxia are shown. (*E*) The percentage of HIF1α^+^ cells in PyMT1/GPx2 KD relative to PyMT1 control cells cultured under normoxia or hypoxia, was gauged by the number of HIF1α nuclear-stained cells; mean ± SEM; *P < 0.05, **P < 0.01, ns = *P* > 0.05. (*F*) PyMT1 control, PyMT1/GPx2 KD cells (sh3 and sh4) were treated with ± 5 mM NAC or 5 nM echinomycin overnight under normoxia or hypoxia; RNA from four replicas each was tested for VEGFA mRNA by qRT-PCR; mean ± SEM; **P < 0.01, *P < 0.05; ns = *P* > 0.05. (*G*) Mice bearing PyMT1/GPx2 KD tumors at 64-mm^3^ size (*n* = 6) were injected daily intraperitoneally with vehicle (DMSO) or 10 μg/kg of echinomycin/DMSO for 21 d; tumor growth curves are shown as mean ± SEM, *P < 0.05. (*H*) Vehicle or echinomycin-treated GPx2 KD tumors from three mice were immunoblotted for VEGFA, GLUT1, or actin. (*I*) Sections (12 total) from each GPx2 KD tumor treated with vehicle or echinomycin were costained for endomucin (FITC) and laminin (TRITC). (*J*) The fraction of vessels with laminin/endomucin colocalization (maturation) was quantified by ImageJ; mean ± SEM; ***P < 0.001. (*K*) Images from H&E-stained sections and 8-OxodG–stained sections from echinomycin- and vehicle-treated tumors are shown. (*L* and *M*) Quantification of three sections from three mice each per each condition shows the percentage hypoxic areas (*L*) and 8-OxodG^+^ cells (*M*) per section; mean ± SEM; **P < 0.01, ****P < 0.0001.

Importantly, to determine whether the biological effects caused by GPx2 loss were not simply due to tumor cell hyperproliferation causing crowding and hypoxia, we analyzed these effects in similarly-sized GPx2 KD and control tumors (*SI Appendix*, Fig. S4*A*). In this setting, GPx2 KD tumors exhibited a consistent phenotype by increasing HIF1α and VEGFA expression (*SI Appendix*, Fig. S4 *B* and *C*), resulting in increased cell proliferation, angiogenesis, and hypoxia (*SI Appendix*, Fig. S4 *D* and *E*). Moreover, GPx2 KD induced striking lung metastasis, which was observed 8 wk postsurgical excision of similarly sized primary tumors (*SI Appendix*, Fig. S4*F*). Altogether, these data confirmed that GPx2 KD actively promotes key biological changes, causing malignancy via ROS signaling leading to vascular modulation and hypoxia.

### GPx2 Loss Causes Abnormal Angiogenesis Due to Impaired Vasculature Causing Poor Perfusion.

It was paradoxical that despite increasing vessel density, GPx2 KD caused highly hypoxic tumors. This suggested that GPx2 loss impairs the vasculature by causing insufficient oxygen delivery to the tumor (26, 27), which was consistent with the dense and tortuous vasculature observed in GPx2 KD tumors. To determine whether vascular abnormality was due to poor vessel perfusion, we used retro-orbital injection of lectin-TRITC, a single subunit glycoprotein that binds with high affinity to endothelial cell membranes under normal perfusion (28). Interestingly, compared to the regularly shaped PyMT tumor vessels, which displayed colocalized lectin-TRITC with endomucin-FITC in most vessels (*SI* *Appendix*, Fig. S5*A*), GPx2 KD tumor vessels were devoid of lectin-TRITC at the endothelial cell membrane (*SI Appendix*, Fig. S5*A*). The perfusion ratio or the fraction of endomucin-FITC^+^ vessels colocalizing with lectin-TRITC, was significantly reduced in GPx2 KD tumors relative to controls (*SI Appendix*, Fig. S5*B*), suggesting GPx2 KD in tumor cells impairs vascular perfusion. To further determine whether hypoperfusion was caused by impaired vessel maturation due to defects in endothelial basement membrane assembly (28), we costained tumors for endomucin and laminin to visualize vascular endothelial basement membranes. While laminin (TRITC) was colocalized with endomucin (FITC) in a large fraction of PyMT1 tumor vessels (*SI Appendix*, Fig. S5*C*), it was severely impaired in GPx2 KD tumors, with fewer vessels staining for both markers (*SI Appendix*, Fig. S5 *C* and *D*).

Interestingly, VEGFA overproduction was also shown to cause vascular malfunction via disruption of PDGFRβ signaling in vascular smooth muscle cells, thereby preventing pericyte coverage of vascular sprouts (14). We therefore stained tumors for endomucin and PDGFRβ, which mark vessels and pericytes, respectively. While PyMT1 control tumors contained PDGFRβ^+^ pericytes nearby endomucin labeled vessels in (*SI Appendix*, Fig. S5*E*), GPx2 KD tumors contained a much lower percentage of vessels covered by pericytes (*SI Appendix*, Fig. S5 *E* and *F*). These data demonstrate the effects of GPx2 loss might promote vascular malfunction by impairing endothelial basement membrane assembly and pericyte coverage, resulting in poor perfusion, and hence hypoxia.

### A Signaling Pathway Driving ROS-Mediated HIF1α Stabilization Leading to VEGFA Up-Regulation.

We sought to elucidate how GPx2 loss modulates the vasculature. Consistent with angiogenesis, VEGFA was expressed at high levels in GPx2 KD tumors relative to controls, as shown by immunoblotting (Fig. 3 *A* and *B*) and immunostaining (Fig. 3*C*). An identical pattern was observed for HIF1α, a known transcriptional regulator of VEGFA (Fig. 3 *A, B,* and *C*). These data were further validated in vitro, demonstrating substantial increases in HIF1α protein in PyMT1/GPx2 KD cells relative to control cells, grown under hypoxia (1% oxygen) (Fig. 3 *D* and *E*), and to a lesser extent, also under normoxia (20% oxygen) (Fig. 3*E*). We next tested whether GPx2 loss up-regulates HIF1α due to protein stabilization by ROS (9, 10). In support of this idea, treatment of GPx2 KD cells with *N*-acetylcysteine (NAC), a thiol antioxidant that quenched ROS (*SI Appendix*, Fig. S3*F*), prevented GPx2 KD-stimulated up-regulation of HIF1α (Fig. 3*E*). We next tested whether GPx2 KD affects VEGFA expression; indeed, we found that GPx2 KD caused a four- and eightfold increase in VEGFA mRNA in cells grown under hypoxia and normoxia (two- and fourfold) (Fig. 3*F*). To determine whether VEGFA up-regulation by GPx2 KD was due to HIF1α stabilization by ROS, we tested whether inhibition of ROS or HIF1α with NAC or echinomycin, respectively (29), affects VEGFA expression. Interestingly, treatment of GPx2 KD cells with either drug blocked the increase in VEGFA mRNA in GPx2 sh3 and sh4 KD cells relative to control cells (Fig. 3*F*), cultured under normoxia or hypoxia. These data support the notion that GPx2 loss up-regulates VEGFA via HIF1α stabilization by ROS, which causes poor perfusion and consequently hypoxia, thereby potentiating HIF1α signaling.

**Fig. 4.**
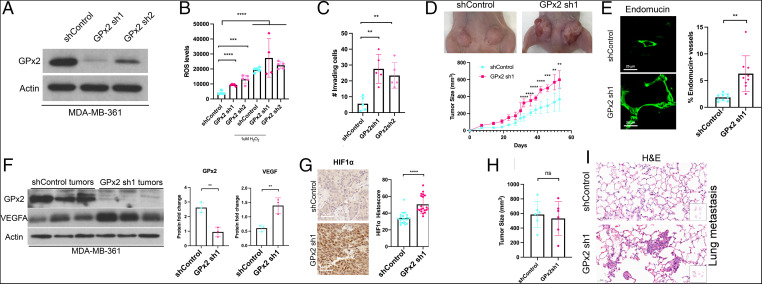
GPx2 loss of function in human BC cells confirms regulation of tumor growth, angiogenesis, HIF1α, and VEGFA by ROS. (*A*) MDA-MB-361 control and GPx2 KD cells (sh1 and sh2) were immunoblotted with anti GPx2 or actin antibody. (*B*) ROS levels were measured in sh1, sh2 cells and control cells untreated or treated with 1 μM H_2_O_2_; mean ± SEM; ***P < 0.001, ****P < 0.0001. (*C*) The number of GPx2 sh1 and sh2 cells relative to control cells invading Matrigel is shown as mean ± SEM; **P < 0.01. (*D*) MDA-MB-361 control and GPx2 sh1 cells (4 × 10^6^) were each injected into two mammary fat pads of female athymic nude mice (*n* = 3), shown by representative mice. Tumor growth (tumor volume) over 53 d after tumor onset; mean ± SEM; **P < 0.01, ***P < 0.001, ****P < 0.0001. (*E*) Six tumors from MDA-MB-361 control and GPx2 sh1 were stained for endomucin (FITC). The fraction of endomucin-labeled vessels per section (three sections each) were quantified by ImageJ; mean ± SEM; **P < 0.01. (*F*) MDA-MB-361 control and GPx2 sh1 tumors (*n* = 3) were immunoblotted for GPx2, VEGFA, and actin; protein levels in each condition are shown as bar graphs, mean ± SEM; **P < 0.01. (*G*) HIF1α levels in MDA-MB-361 control versus GPx2 sh1 tumors (*n* = 6, 2 sections each) were quantified by histoscore; mean ± SEM; ****P < 0.0001. (*H*) Similarly sized tumors (*n* = 6) from 3 mice injected with either MDA-MB-361 control or GPx2 sh1 cells (4 × 10^6^) were surgically removed when tumors were 10 mm in diameter (28 d for control and 21 d for GPx2 sh1). Bar graphs display tumor volume at end point; mean ± SEM; ns = *P* > 0.05. (*I*) H&E staining of lung sections from whole lung lobes (boxes) at 8 wk post surgery shows metastatic foci in two of three mice.

These data suggested that inhibition of HIF1α signaling might renormalize vessels and shrink the tumor. Indeed, treatment of mice bearing PyMT1/GPx2 KD tumors with daily intraperitoneal injection of echinomycin for 21 d following tumor onset substantially reduced mammary tumor growth (Fig. 3*G*). Immunoblotting of tumors confirmed inhibition of VEGFA or GLUT1, two HIF1α target genes, in echinomycin-treated GPx2 KD tumors relative to vehicle treated tumors (Fig. 3*H*). Interestingly, staining tumors for endomucin and laminin revealed a marked increase in colocalization of both proteins in vessels from echinomycin-treated tumors relative to controls (Fig. 3 *I* and *J*). Moreover, echinomycin caused reduced hypoxia and ROS-induced DNA damage in tumors (Fig. 3 *K*–*M*). These data underscore the notion that echinomycin restores vascular function by inhibiting HIF1α/VEGFA axis due to ROS up-regulation by GPx2 loss.

### GPx2 Loss- and Gain-of-Function in Human BC Cells Confirm Stark Tumor-Suppressive Effects.

To confirm the effects of GPx2 on human BC, we knocked down GPx2 in the human MDA-MB-361 cell line, which expresses GPx2 and partially resembles the PyMT model in that it expresses estrogen receptor (ER) and HER2. Conversely, we overexpressed GPx2 in the triple-negative and metastatic MDA-MB-231 cell line.

KD of GPx2 in MDA-MB-361 cells by two independent shRNAs (Fig. 4*A*) stimulated increases in ROS levels and Matrigel invasion (Fig. 4 *B* and *C*), as well as in tumor growth in vivo (Fig. 4*D*). Moreover, GPx2 KD resulted in abnormal intratumoral vessels that were long and tortuous (Fig. 4*E*), which were consistent with HIF1α and VEGFA up-regulation in GPx2 KD in mammary tumors (Fig. 4 *F* and *G*). Furthermore, we tested the effect of GPx2 KD spontaneous lung colonization, 8 wk postremoval of similarly sized MDA-MB-361 control and GPx2 KD mammary tumors (Fig. 4*H*), to uncouple metastasis from tumor burden. GPx2 KD was able of stimulating metastasis in two of the three mice analyzed (Fig. 4*I*), implying a trend toward metastatic progression that warrants validation in a larger mouse sampling.

**Fig. 5.**
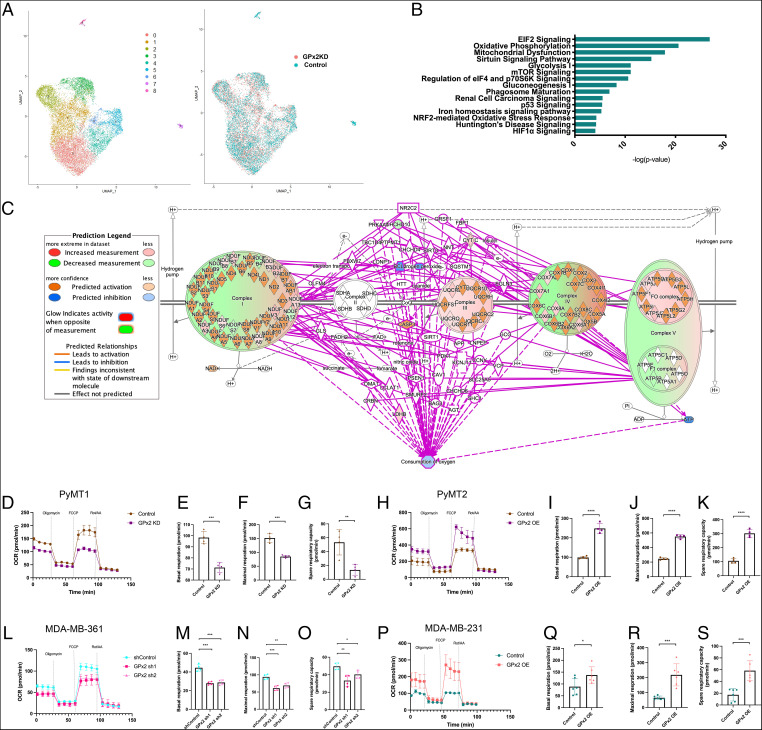
GPx2 regulates OXPHOS revealed by scRNA-seq and metabolic testing. (*A*) UMAP projection of comprehensively integrated clustering results from one PyMT1 control and one PyMT1/GPx2 KD tumor revealed seven epithelial clusters (cluster 0, 1, 2, 3, 4, 5, and 6) and two stromal clusters (cluster 7 and 8). UMAP shows the overlap between cell clusters of GPx2 KD (red) and control (green) tumors. (*B*) The genes that were differentially expressed between GPx2 KD and control tumor cells, regardless of clusters or cell states, were analyzed using IPA; over-represented signaling pathways by GPx2 KD are indicated by -log(*P* value). (*C*) Overlay of the differentially expressed genes regulated by GPx2 KD onto OXPHOS pathway in the Ingenuity Knowledge Base predicted inhibition of oxygen consumption (blue circle). (*D*) PyMT1/GPx2 KD and PyMT1 control cells were assayed for OCR; normalized OCR data comparing both groups were derived for each of the mitochondrial respiration steps after 1 μM oligomycin, 1 μM FCCP, and 0.5 μM rotenone/antimycin treatment. (*E*–*G*) Basal respiration, maximal respiration, and spare respiratory capacity are shown as mean of four replicas ± SEM; ***P < 0.001; **P < 0.01. (*H*) PyMT2/GPx2 OE cells were compared to PyMT2 control cells for OCR. (*I*–*K*) Basal respiration, maximal respiration, and spare respiratory capacity are shown as mean of four replicas ± SEM; ****P < 0.0001. (*L*–*O*) MDA-MB-361/GPx2 sh1 and GPx2 sh2 cells were compared to control cells for OCR; basal respiration, maximal respiration, and spare respiratory capacity are shown as mean of four replicas ± SEM; *P < 0.05, **P < 0.01, ***P < 0.001. (*P*–*S*) MDA-MB-231/GPx2 OE cells were compared to control cells for OCR; maximal respiration, and spare respiratory capacity are shown as mean of six replicas ± SEM; ***P < 0.001, *P < 0.05.

In contrast, OE of GPx2 in MDA-MB-231 cells attenuates ROS, proliferation, and invasion in vitro (*SI Appendix*, Fig. S6 *A*–*D*). Moreover, GPx2 OE dramatically inhibits mammary tumor growth and spontaneous lung metastasis (*SI Appendix*, Fig. S6 *E*–*F*). GPx2 OE in tumors led to reduced intratumoral vessel density and HIF1α levels (*SI Appendix*, Fig. S6 *G–I*), confirming the effect of GPx2 on vascular modulation and hypoxia. Collectively, our data demonstrate a dramatic effect of redox signaling by GPx2 loss on metastatic progression of human BC xenografts representative of luminal B, HER2-enriched, and TNBC subtypes.

### scRNA-Seq Supports ROS/HIF1α/VEGFA Pathway Activation by GPx2 Loss.

In light of the striking effects of GPx2 on the malignant phenotype, we sought to examine the effect of GPx2 loss on tumor heterogeneity at the single-cell level to identify potential cell populations (i.e., clusters) that might drive BC progression. We performed scRNA-seq of GFP-labeled PyMT1/GPx2 KD and PyMT1 control cells, freshly isolated from one mammary tumor each (30). We performed clustering analysis of the cells and used uniform manifold approximation and projection (UMAP) to visualize the clusters shared by the GPx2 KD and control tumor (31). The clusters were defined by cells with similar variation in gene expression and were grouped into seven epithelial clusters (clusters 0, 1, 2, 3, 4, 5, and 6) and two nonepithelial clusters (clusters 7 and 8) (Fig. 5*A*) that might be tumor-associated stromal cells.

**Fig. 6.**
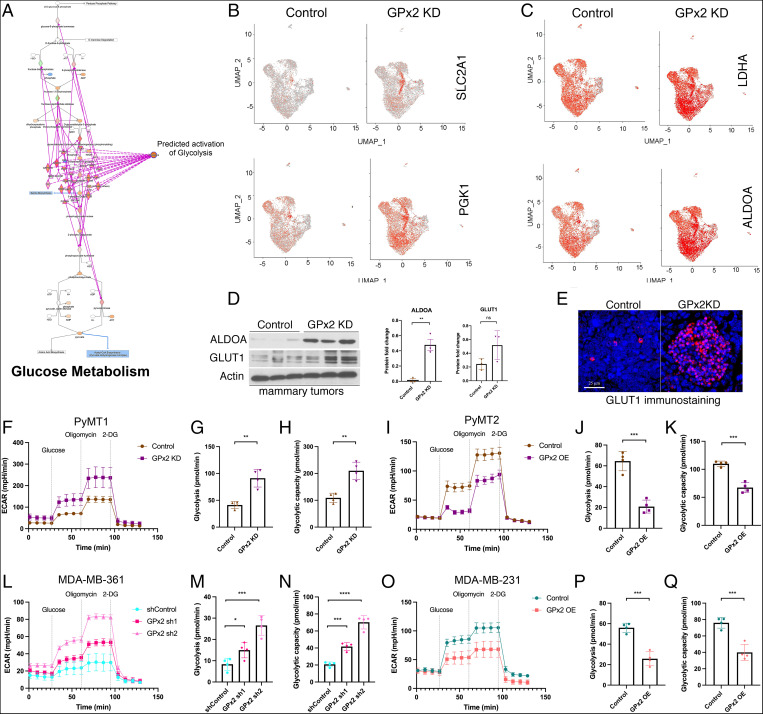
GPx2 loss stimulates glycolysis in vitro and in vivo. (*A*) Overlay of the differentially expressed genes regulated by GPx2 KD to glycolysis pathway in the Ingenuity Knowledge Base predicts activation of glucose metabolism. (*B* and *C*) Feature plots in low-dimensional space showed increased expression of SLC2A1, PGK1, LDHA, ALDOA glycolytic genes in the GPx2 KD relative to control tumor. (*D*) Western blots of ALDOA, GLUT1, and actin in PyMT1/GPx2 KD vs. control tumors. Graphs of densitometric values of ALDOA and GLUT1 immunoblots relative to actin are shown as mean ± SEM; **P < 0.01 for ALDOA. (*E*) PyMT1 control and PyMT1/GPx2 KD tumors (five each) were immunostained with anti-GLUT1 antibody followed by TRITC detection and DAPI counterstain; a representative image of each tumor is shown. (*F*) PyMT1/GPx2 KD and PyMT1 control cells were assayed for ECAR; normalized ECAR values were derived after sequential treatment with 20 mM glucose, 1 μM oligomycin, and 50 mM 2-DG; (*G* and *H*) glycolysis and glycolytic capacity are shown as mean of four replicas ± SEM; **P < 0.01. (*I*–*K*) PyMT2/GPx2 OE cells and PyMT2 control were assayed for ECAR; glycolysis and glycolytic capacity are shown as mean of four replicas ± SEM; ***P < 0.001. (*L*–*N*) MDA-MB-361/GPx2 sh1 and GPx2 sh2 cells were compared to control cells for ECAR; glycolysis and glycolytic capacity are shown as mean of four replicas ± SEM; *P < 0.05, ***P < 0.001, ****P < 0.0001. (*O*–*Q*) MDA-MB-231/GPx2 OE cells were compared to control cells for ECAR; glycolysis and glycolytic capacity are shown as mean of four replicas ± SEM; ***P < 0.001.

Our scRNA-seq data confirmed a fivefold reduction in GPx2 mRNA in the GPx2 KD tumor (*SI Appendix*, Fig. S7 *A* and *B*) and violin plots confirmed a dramatic increase in HIF1α and VEGFA mRNAs in most of the clusters that make up the GPx2 KD tumor (*SI Appendix*, Fig. S7 *C* and *D*). To identify the overrepresented upstream regulators that may explain the GPx2 KD-stimulated changes in gene expression, we analyzed the top 583 genes that were significantly differentially expressed in the GPx2 KD relative to the control tumor, using Ingenuity Pathway Analysis (IPA). This bulk analysis did not take into account the clusters or cell states in which these genes were expressed differentially. Interestingly, we identified the HIF signaling pathway as a pivotal upstream regulator of the GPx2 KD tumor response, as shown by activation of HIF1α (Z-score = 2.033) and HIF1β (ARNT) (Z-score = 3.065) that was interestingly accompanied by inhibition of PHD (EGLN) (Z-score = −2.455) (*SI Appendix*, Fig. S7 *E*–*G*), a destabilizer of HIF1α. Next, to determine the type of disease that might be controlled by the upstream regulators and their target genes, we performed the regulator effect analysis by IPA, which integrates the overrepresented upstream regulators with the differentially expressed genes by GPx2 KD. This led to the prediction of disease and function model linking mammary tumorigenesis (Z-score = 2.94) to angiogenesis (Z-score = 2.94), which was based on the highest consistency scores among all candidate disease models (*SI Appendix*, Fig. S7 *H*–*J*). Of note, overrepresented pathway analysis revealed that the GPx2 KD tumor was enriched in key signaling pathways including EIF2 signaling, OXPHOS, mitochondrial dysfunction, Sirtuin signaling, glycolysis, and mammalian target of rapamycin (mTOR) signaling, among others (Fig. 5*B*), which likely form a coordinated adaptive response to oxidative stress and hypoxia elicited by GPx2 loss.

Importantly, analysis of the differentially expressed genes in the GPx2 KD tumor relative to control tumor revealed changes in mRNA expression of other members of the GPx family. This included a 1.4-fold increase in GPx1 expression (log fold-change [FC] = 0.5) and a 0.36-fold reduction in GPx4 expression (logFC = −0.6), whereas GPx3 and GPx5 mRNA expression was undetected (*SI Appendix*, Fig. S8*A*). In addition, the expression of PRDX2 and PRDX5 in the GPx2 KD tumor was increased by 1.3-fold (logFC = 0.4) and catalase by 1.2-fold (logFC = 0.27), whereas the expression of SOD2 was unchanged (*SI Appendix*, Fig. S8*A*). These data support a predominant role for GPx2 KD in regulating the tumor response; however, they do not rule out that mild increases in PRDX2 or PRDX5 do not contribute to tumor aggressiveness, especially since these antioxidants were shown to be associated with worse patient prognosis (32).

### GPx2 Loss Suppresses OXPHOS and Stimulates Aerobic Glycolysis.

HIF1α is up-regulated in cells undergoing hypoxia, where it inhibits OXPHOS to lessen dependency on oxygen while preventing excessive ROS production by the mitochondria to cause cytotoxicity (33). It remained to be determined whether oxidative respiration was negated by GPx2 KD. Overlay of the differentially expressed genes by GPx2 KD onto the genes underlying OXPHOS in the Ingenuity Knowledge Base led IPA to predict that oxygen consumption in the GPx2 KD tumor was in fact inhibited (Fig. 5*C*). To test this projection, we measured oxygen consumption rate (OCR) in cell lines using the Seahorse XF Cell Mito Stress Test (Fig. 5 *D*–*S*). This assay is able to test distinct features of mitochondrial respiration involving basal respiration, ATP production, proton leak, maximal and spare respiratory rates, as well as nonmitochondrial respiration. Indeed, PyMT1/GPx2 KD tumor cells exhibited striking changes in OCR that led to stark reductions in basal respiration, maximal respiration, and spare respiratory capacity relative to control cells (Fig. 5 *D*–*G*). A similar trend was noted in vivo showing lower OCR in PyMT1/GPx2 KD tumors relative to PyMT1 control tumors (*SI Appendix*, Fig. S8*B*). Conversely, GPx2 OE in PyMT2 cells enhanced OCR (Fig. 5*H*), as indicated by increased basal, maximal, and spare respiratory capacity (Fig. 5 *I*–*K*). Importantly, these data were reciprocated in human BC cells. Namely, silencing GPx2 with sh1 or sh2 hairpins in MDA-MB-361 cells yielded substantially reduced levels of basal, maximal, and spare respiration levels relative to control cells (Fig. 5 *L*–*O*). Furthermore, GPx2 OE in MDA-MB-231 cells stimulated noted increases in all of the above mitochondrial respiration steps (Fig. 5 *P*–*S*). Hence, these data underscore a clear inhibition of OXPHOS by GPx2 loss, which is likely due to increased ROS production.

Stabilization of HIF1α has been shown to drive the Warburg effect, which shifts ATP production from OXPHOS to aerobic glycolysis (34). Annotation of the differentially expressed genes by GPx2 KD onto the genes regulating glycolysis in the Ingenuity Knowledge Base predicted enhanced glycolysis in the GPx2 KD tumor (Fig. 6*A*). These data were consistent with UMAP plots showing dramatically higher levels of glycolytic gene transcripts in the GPx2 KD tumor. Indeed, the GPx2 KD tumor expressed notably higher levels of glucose transporter GLUT1 (SLC2A1), aldolase-A (ALDOA), phosphoglycerate kinase (PGK1), and lactate dehydrogenase A (LDHA) than in the control tumor (Fig. 6 *B* and *C*). These data were confirmed by Western blots showing increases in ALDOA and GLUT1 protein in GPx2 KD tumors (Fig. 6*D*). Furthermore, immunostaining of tumors revealed GLUT1 up-regulation in hypoxic pockets, likely due to HIF1α signaling that promotes a glycolytic switch in the tumor (Fig. 6*E*).

**Fig. 7.**
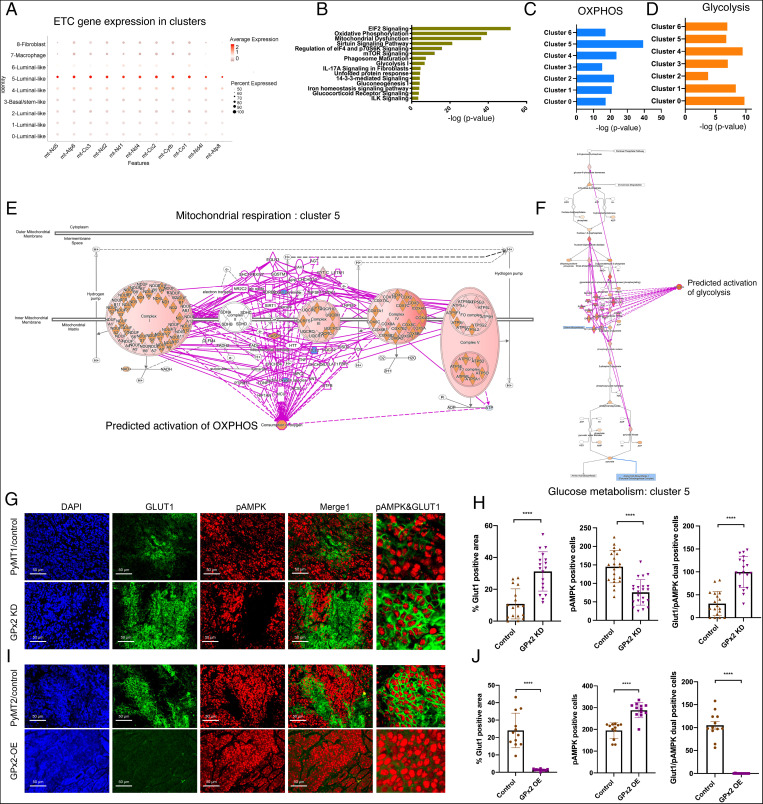
GPx2 KD stimulates a tumor cell cluster with a hybrid metabolic phenotype. (*A*) Dot plot of overrepresented electron transport chain (ETC) genes shows cluster 5-specific expression across nine clusters. Red dot intensity represents average expression of mRNA, and diameter of the dot refers to the percentage of cells expressing the indicated gene in the *x* axis. (*B*) Core analysis using IPA of differentially expressed genes in cluster 5 revealed over-represented pathways that were enriched by GPx2 KD. (*C* and *D*) Comparing OXPHOS and glycolysis enrichment across all clusters. (*E* and *F*) Overlay of the differentially expressed genes regulated by GPx2 KD in cluster 5 onto the OXPHOS and glycolysis pathway in the Ingenuity Knowledge Base predicted activation of both oxygen consumption/OXPHOS and glycolysis. (*G* and *I*) PyMT1 and PyMT1/GPx2 KD mammary tumors from five mice each; PyMT2 and PyMT2/GPx2 OE tumors from four mice each were sectioned and three random sections from each tumor were coimmunostained with anti GLUT1 (FITC) and p-AMPK (TRITC) antibody and counterstained with DAPI (blue); the far right panels represent blown images to highlight the pAMPK/GLUT1^+^ cells. (*H* and *J*) The fraction of GLUT1 positive tumor area per section was quantified by ImageJ (*Left*); mean ± SEM; the number of p-AMPK^+^ tumor cells (*Center*) and the number of GLUT1/p-AMPK dual positive tumor cells (*Right*) per section were calculated as mean ± SEM. Two-tailed *t* test; ****P < 0.0001.

To confirm the effect of GPx2 KD on glycolysis, we measured the extracellular acidification rate (ECAR), that results from pyruvate conversion into lactate, in PyMT1/GPx2 KD relative to control PyMT1 cells (Fig. 6 *H*–*Q*). Indeed, the glycolytic rate and capacity were both augmented in PyMT1/GPx2 KD cells relative to control cells (Fig. 6 *G* and *H*), a metabolic trend that was reversed by GPx2 OE in PyMT2 cells (Fig. 6 *I*–*K*). Finally, these results were reproduced in human BC cells, showing higher glycolytic activity in MDA-MB-361/GPx2 KD cells (Fig. 6 *L*–*N*), and opposite effects in MDA-MB-231/GPx2 OE cells (Fig. 6 *O*–*Q*). In addition, in support of ROS as a mediator of the shift from OXPHOS to glycolysis, we found that treatment of PyMT1/GPx2 KD cells with NAC increased OCR and decreased ECAR (*SI Appendix*, Fig. S8 *C* and *D*). Hence, GPx2 KD promotes a glycolytic switch that drives the bulk of the tumor.

### GPx2 KD Stimulated a Tumor Cell Cluster with a Hybrid Metabolic Phenotype.

We next investigated whether metabolic reprogramming by GPx2 KD was uniform in all the tumor cell clusters. Remarkably, of all seven clusters, cluster 5 gene expression was, regardless of GPx2, overrepresented with genes associated with the mitochondrial electron transport chain, implying this cluster regulates metabolic activity (Fig. 7*A*). Of note, by inputting the differentially expressed genes by GPx2 KD in cluster 5 into IPA, we identified overrepresented pathways in this cluster that were similar to those in the bulk of the tumor, including OXPHOS and glycolysis (Fig. 7*B*). Moreover, analysis of each of the tumor cell clusters showed that GPx2 KD caused cluster 5 to be highly enriched in OXPHOS genes relative to all other clusters, as judged by the -log (*P* value) (Fig. 7*C*), relative to moderate enrichment in glycolysis (Fig. 7*D*). Remarkably, overlay of cluster 5-specific differentially expressed genes in OXPHOS or glycolysis pathways in the Ingenuity Knowledge Base predicted that oxygen consumption and glycolysis were both activated by GPx2 KD in this cluster (Fig. 7 *E* and *F*). These data were further corroborated by unbiased analysis of the differentially expressed OXPHOS- and glycolysis-associated genes by GPx2 KD in cluster 5, showing unequivocal increases in a large number of genes regulating OXPHOS or glycolysis (*SI Appendix*, Fig. S9*A* ). In contrast to cluster 5, GPx2 KD stimulates the Warburg effect in all other tumor cell subpopulations (clusters 0, 1, 2, 3, 4, and 6) as shown by inhibited OCR and enhanced glycolysis (*SI Appendix*, Fig. S10). These data demonstrate that GPx2 KD promotes aerobic glycolysis in most of the tumor, while endowing a tumor cell subpopulation represented by cluster 5 with the ability to use both OXPHOS and glycolysis.

Our data are in agreement with previous studies demonstrating hybrid metabolism in solid tumors. These findings were based on computational modeling of tumor metabolism based on HIF1α and AMPK gene signatures that were derived from RNA-seq data of bulk tumor and single cells from several tumor types (17, 35). Both AMPK and HIF1α promote glucose uptake, thereby stimulating OXPHOS and glycolysis, respectively. Whereas AMPK regulates glucose and fatty acid oxidation to generate Acetyl Co-A from pyruvate for OXPHOS, HIF1α stimulates glycolysis to convert pyruvate into lactate (17, 36).

To confirm the dual metabolic state of cluster 5, GPx2 KD tumors were compared to control tumors for HIF1α and AMPK activities. We measured GLUT1 expression and AMPK phosphorylation as surrogates for HIF1α and AMPK activation, respectively. Overall, GPx2 KD tumors contained a significantly higher percentage of cells expressing GLUT1, and a lower percentage of cells expressing p-AMPK relative to control tumors (Fig. 7 *G* and *H*), consistent with the Warburg effect in the bulk of the tumor. However, GPx2 KD tumors were enriched in discrete areas coexpressing GLUT1 and p-AMPK relative to control tumors (Fig. 7*G*, last panels, and Fig. 7*H*), implying that GPx2 KD supports a hybrid metabolic phenotype. Of note, these metabolic changes, indicated by GLUT1 and p-AMPK expression, were maintained in similarly sized tumors, thus supporting effects that may be due to increases in tumor bulk by GPx2 KD (*SI Appendix*, Fig. S4*G*). Conversely, GPx2 OE PyMT2 tumors were GLUT1^−^ but contained a higher level of p-AMPK^+^ cells than PyMT2 controls (Fig. 7 *I* and *J*), which is consistent with the higher OCR of these cells. In line with these observations, GPx2 OE PyMT2 tumors had dramatically fewer hybrid areas relative to control tumors (Fig. 7*I*, last panels, and Fig. 7*J*). We further validated these data in human BC cells, showing that the fraction of cells expressing p-AMPK and GLUT1 was increased upon GPx2 KD in MDA-MB-361 cells (*SI Appendix*, Fig. S11 *A*–*D*), and decreased upon GPx2 OE in MDA-MB-231 cells (*SI Appendix*, Fig. S11 *E*–*H*). These data demonstrate that GPx2 loss regulates a bioenergetically hybrid cell population that might fuel progression toward metastasis.

### GPx2 KD Stimulated a Hybrid Metabolic Cluster Endowed with an EMT/Stem-Like Gene Signature.

In light of these findings, we sought to investigate whether the GPx2 KD tumor was enriched in EMT or stemness genes that may be associated with metabolic plasticity of cluster 5. We firstly analyzed the overall differential expression of genes in the GPx2 KD tumor relative to control tumor. This revealed an enrichment of mesenchymal/stem-like genes (KRT14, LGALS7, LGALS3, S100A6, COL9A3, MMP2, KLF9, KLF10, RAN) and down-regulation of epithelial/luminal genes (EPCAM and CLDN7) (*SI Appendix*, Fig. S9*B*), which is consistent with an EMT or stem-like phenotype.

To further investigate the functional identity of cluster 5, we unbiasedly analyzed the differentially expressed genes between cluster 5 and all other clusters. This showed that, regardless of GPx2, cluster 5 was enriched in a signature comprised of mesenchymal/stem-like genes (FGFR1, JUN, SOX9, NOTCH1, STAT3, FOXC1, ITGA6, FOXP1), and devoid of epithelial/luminal genes (EPCAM, CLDN3, and CLDN7) (*SI Appendix*, Fig. S9*C*). Next, we compared cluster 5 gene expression in the PyMT1/GPx2 KD tumor to that in the control tumor. This revealed that GPx2 KD further enriched cluster 5 in basal/mesenchymal-like genes (KRT14, ITGA6, FGFR1, COLA9A3, S100s) relative to cluster 5 in control tumor (*SI Appendix*, Fig. S9*D*). However, GPx2 KD also increased the expression of luminal lineage genes (KRT8 and KRT18) in cluster 5 (*SI Appendix*, Fig. S9*D*). Interestingly, cluster 5/GPx2 KD gene expression was also enriched in galectin genes (LGALS1, LGALS3, LGALS7) but was devoid of GSK3β expression (*SI Appendix*, Fig. S9*D* ). Galectins may be associated with antiapoptotic activity and therapeutic resistance, whereas low GSK3β activity may reflect activation of Wnt signaling, two features normally associated with cancer stem cells (37–39). These data raise the possibility that cluster 5 may play a role in cancer stemness and metastasis that is further exacerbated by GPx2 dysregulation.

## Discussion

Our study points to an important and unresolved conundrum in cancer biology: whether redox regulation by antioxidant molecules promotes or inhibits tumorigenesis. In contrast to our data supporting a tumor-suppressive function of GPx2, other studies have pointed to protumorigenic effects (40), implying GPx2 exerts complex biological functions. As in breast, GPx2 loss in bladder and esophageal carcinomas led to disease progression and worse prognosis (41, 42). Moreover, GPx2 knockout in mice resulted in intestinal tumorigenesis and sensitized skin to cancer by irradiation (40). In contrast, GPx2 OE in several carcinoma types was associated with poor prognosis (43). To reconcile these opposites, GPx2 expression was shown to be tumor stage-dependent, as its expression was transiently up-regulated in early colon and lost during late-stage tumorigenesis (43). We suspect GPx2 up-regulation in early-stage carcinomas might protect tumor cells from the effects of ROS on oncogenic signaling, leading to neoplastic progression. In support of this idea, a study of pancreatic cancer demonstrated a differential regulation of tumor initiation versus metastatic progression by TIGAR, an antioxidant gene. This study showed that deletion of TIGAR inhibits the development of premalignant lesions while promoting metastatic spread (44). These findings highlight the complexity of ROS regulation in cancer progression, cautioning against the use of random antioxidant therapy in cancer patients.

In this study, we modeled the effect of GPx2 loss- and gain-of-function on the mammary tumor phenotype using a vast array of BC models representing most of the molecular subtypes including luminal B, HER2-enriched, and TNBCs. Our findings demonstrate profound effects of GPx2 on BC progression, that is consistent with clinical correlations between GPx2 loss, disease progression, and poor patient survival. GPx2 loss provoked striking ROS signaling despite expression of GPx1, PRDX2, PRDX5, and catalases. These antioxidants may serve in keeping ROS below the lethal threshold, while maintaining pro-oncogenic redox signaling. Of note, PRDXs were found to be associated with poor patient survival (32), thus pointing to unresolved questions about the basis for the pro- or antitumorigenic effect of antioxidants.

Our cumulative data support a view that redox signaling by GPx2 loss activates ROS/HIF1α/VEGFA signaling, resulting in abnormal angiogenesis, thus exacerbating hypoxia and HIF1α signaling, leading to metabolic heterogeneity and malignant progression. The up-regulation of HIF1α in GPx2 KD cells likely occurs via transcriptional and posttranslational regulation, the latter involving mRNA translation via ROS activation of the PI3K-mTOR pathway (45). This was supported by IPA upstream regulator analysis of the GPx2 KD tumor, showing activation of mTOR kinase (Z-score = 2.26) (*SI Appendix*, Fig. S12*A*). Moreover, our data point to inhibition of PHD and Sirtuin signaling (Z-score < −2.0) (*SI Appendix*, Fig. S12*B*), two powerful destabilizers of HIF1α protein (46). Likely, these alterations potentiate HIF1α-mediated transcriptional activation of VEGFA and PDGF, which cause vascular malfunction and hypoxia (14, 47). In fact, treatment of GPx2 KD tumor-bearing mice with echinomycin, a drug that inhibits HIF1α (29), reduced VEGFA expression and tumor growth, while improving vessel maturation (48), underscoring the effect of GPx2 loss on vascular malfunction. Partial vessel normalization by echinomycin might be due to incomplete drug perfusion due to residual impaired vessels in the tumor. Otherwise, ROS may activate novel signaling, which converges with HIF1α to potentiate oncogenesis, a notion that was supported by GSEA of BC datasets, indicating a link between GPx2 underexpression and NF-κB signaling (49).

It is believed that highly proliferating cancer cells rely mostly on glycolysis and less on OXPHOS, to generate both ATP and building blocks for biomass biosynthesis, even under normoxic conditions, known as aerobic glycolysis or the Warburg effect. This notion was supported by our data showing lower OCR and higher ECAR/glycolytic rates by GPx2 KD cells, as well as dramatic up-regulation of GLUT1, ALDOA, PGK1, and LDHA glycolytic genes in GPx2 KD tumors (50). Indeed, these data are supportive of the notion that HIF1α inhibits OXPHOS while it stimulates glycolysis. Namely, HIF1α inhibits pyruvate dehydrogenase kinase, which in turn inactivates pyruvate dehydrogenase that converts pyruvate to acetyl-CoA for OXPHOS. This results in accumulation of pyruvate, which is converted by lactate dehydrogenase into lactate, thus generating NAD^+^ for glycolysis (33).

Interestingly, breast tumors are known to exhibit metabolic heterogeneity with highly metastatic cells exhibiting both OCR and ECAR activities, implying metabolic plasticity conveys a survival advantage (51, 52). Our data show that GPx2 loss endows cluster 5 with the ability to use both OXPHOS and glycolysis, which contrasted with the Warburg effect in all other clusters. In an attempt to identify metabolically hybrid tumor cells, others used mathematical modeling integrating gene expression with metabolic pathways based on AMPK and HIF1α gene signatures, two critical regulators of OXPHOS and glycolysis, respectively (17, 36). Consistent with these findings, we were able to demonstrate higher incidence of OXPHOS/glycolytic cancer cells in the GPx2 KD tumor via dual expression of p-AMPK and HIF1α. Interestingly, tumor cells positive for p-AMPK and GLUT1 were enriched at the interface of normoxic and hypoxic regions of the GPx2 KD tumor, where both metabolic modalities may be needed.

Our data underscore the notion that GPx2 loss drives tumor heterogeneity, resulting in a metabolically hybrid tumor cell population, which in turn activates both AMPK and HIF1α (GLUT1) signaling, likely due to ROS signaling (17, 53). Furthermore, we envision that metabolic heterogeneity drives tumor cell adaptation to various tumor microenvironments. We suspect that tumor cells located in the vicinity of functional blood vessels benefit from OXPHOS relative to cells residing nearby immature vessels or at avascular hypoxic pockets. Conversely, tumor cells migrating away from hypoxic areas into the circulation will be required to rapidly switch from glycolysis to OXPHOS to adapt to increases in oxygen levels in the blood. Finally, upon seeding of distant organs, tumor cells are likely to activate OXPHOS in the lungs, which are highly oxygenated, and glycolysis in the liver, which is low in oxygen (54).

Furthermore, in support of a link between metabolic and phenotypic plasticity, we found that cluster 5 was enriched in an EMT/stem-like signature as compared to all other clusters. Interestingly, GPx2 KD caused cluster 5 to gain expression of mesenchymal- and luminal-like genes, which may in turn drive metabolic heterogeneity. Indeed, others have shown that luminal BCs used OXPHOS, whereas Basal/triple-negative tumors were prone to use glycolysis (55, 56). In addition, cluster 5/GPx2 KD up-regulated S100 genes, known to play a role in metastatic fitness of pancreatic tumors (57), and galectin genes, which may guide therapeutic resistance, and hence tumor recurrence or metastasis (39). These data raise the likelihood of cluster 5 as a proponent of phenotypic and metabolic plasticity, underlying stemness and metastasis. Hence, targeting OXPHOS and glycolysis might eradicate tumors exhibiting a hybrid epithelial/mesenchymal phenotype, known to exhibit higher stem and metastatic activities (58).

Finally, our scRNA-seq showed that GPx2 KD caused dramatic changes in gene expression, indicative of ROS-mediated oxidative stress and hypoxia. Some of the genes involved the HIF1α target genes BNIP3, ROMO-1, and HIGD1A (*SI Appendix*, Fig. S12*C*), which may attenuate potential mitochondrial dysfunction by ROS. Finally, it is noteworthy that EIF2 signaling, which promotes translation initiation, was inhibited in three of the seven clusters in the GPx2 KD tumor (Z-score < −2) (*SI Appendix*, Fig. S10*D*). This is consistent with a potential effect of ROS on endoplasmic reticulum (ER) stress due to misfolding of proteins, which leads to unfolded protein response via inhibition of protein synthesis (59–61).

In sum, the cumulative data point to profound effects of GPx2 loss on mammary tumor progression, resulting in ROS/HIF1α/VEGFA signaling, causing vascular malfunction and hypoxia. This in turn attenuates OXPHOS and activates glycolysis in most of the tumor clusters. However, GPx2 loss drives tumor heterogeneity, thereby selecting for a tumor subpopulation endowed with phenotypic and metabolic plasticity that likely drives malignant progression. Hence the use of drugs targeting OXPHOS and glycolysis may be leveraged to suppress metabolic plasticity, and possibly EMT, stemness, and metastasis. A model summarizing the core and novelty of our findings is displayed in (*SI Appendix*, Fig. S13).

## Materials and Methods

Detailed protocols regarding cell lines and cell culture, animal studies, tumor growth, and lung metastasis, lentivirus production, shRNA lentiviral transduction, GPx2 lentivirus OE, real-time qRT-PCR, antibodies, immunoblotting, immunofluorescence, ROS measurements, cell proliferation, transwell Matrigel migration, immunohistochemistry, vessel perfusion and maturation, in vitro and in vivo oxygen consumption, glycolysis stress test in vitro, cell isolation of mammary tumor cells for scRNA-seq, flow cytometry, library preparation and scRNA-seq, processing of scRNA-seq data, quality control and normalization, integration and clustering, cluster comparison and visualization, differential gene expression, IPA, TCGA data mining, Kaplan–Meier analysis, GSEA, and statistical analysis are described in *SI Appendix*. Animal protocols used for this study were reviewed and approved by the Institute for Animal Studies of the Albert Einstein College of Medicine.

## Supplementary Material

Supplementary File

## Data Availability

The single cell RNA sequencing data reported in this paper have been deposited and publicly released in the Gene Expression Omnibus database, https://www.ncbi.nlm.nih.gov/geo (accession no. GSE152368). Coding analyses for single cell RNA sequencing data are available in GitHub with the URL accession link https://github.com/Malindrie/Breast-cancer-scRNA-seq-analysis. All other study data are included in the main text and *SI Appendix*.
