# Supplementary material for: Redox signaling by glutathione peroxidase 2 links vascular modulation to metabolic plasticity of breast cancer
Source: Proc Natl Acad Sci U S A. 2022 Feb 22;119(8):e2107266119. doi: 10.1073/pnas.2107266119 (PMC8872779; doi:10.1073/pnas.2107266119)
Supplement: Supplementary File [file pnas.2107266119.sapp.pdf]

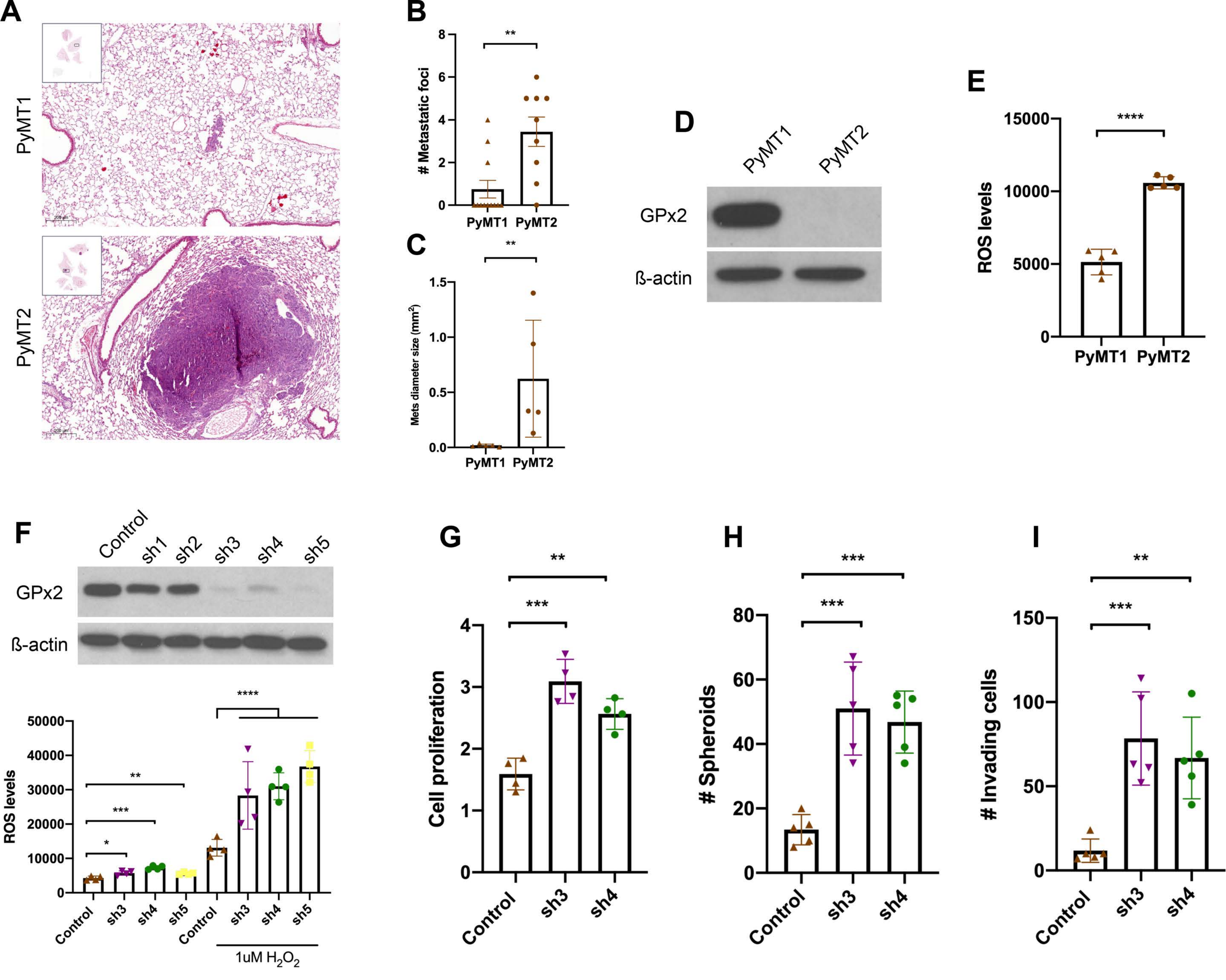
A
PyMT1
PyMT2
B
Metastatic foci
PyMT1
PyMT2
**
C
Mets diameter size (mm2)
PyMT1
PyMT2
**
D
PyMT1
PyMT2
GPx2
ß-actin
E
ROS levels
PyMT1
PyMT2
****
F
Control
sh1
sh2
sh3
sh4
sh5
GPx2
ß-actin
ROS levels
1uM H2O2
G
Cell proliferation
Control
sh3
sh4
H
Spheroids
I
Invading cells

Fig. S1

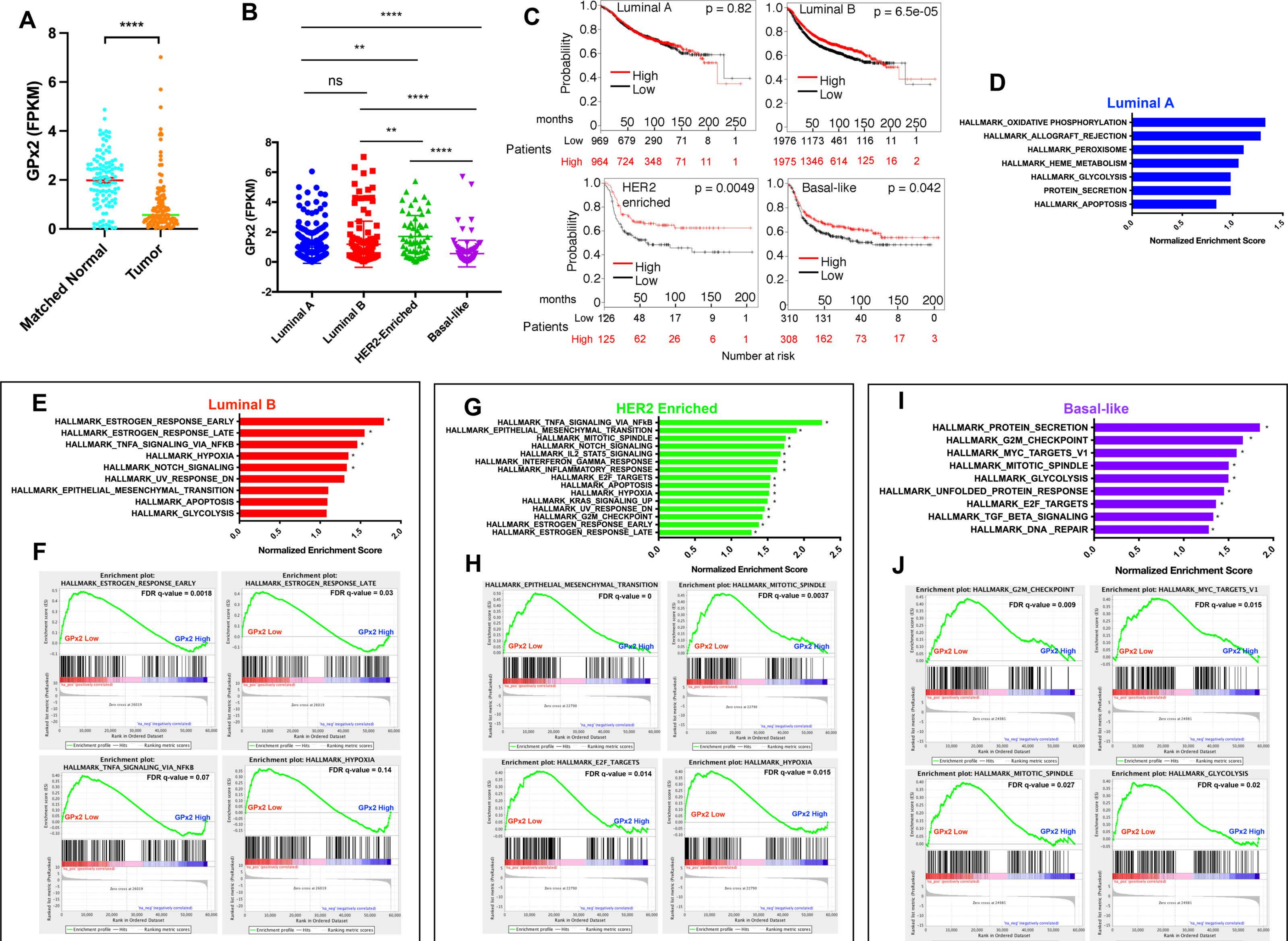
A
GPx2 (FPKM)
****
Matched Normal
Tumor
B
GPx2 (FPKM)
****
**
ns
****
**
****
Luminal A
Luminal B
HER2-Enriched
Basal-like
C
Luminal A p = 0.82
Luminal B p = 6.5e-05
HER2 enriched p = 0.0049
Basal-like p = 0.042
Probability
High
Low
months
Patients
Number at risk
D
Luminal A
HALLMARK_OXIDATIVE PHOSPHORYLATION
HALLMARK_ALLOGRAFT_REJECTION
HALLMARK_PEROXISOME
HALLMARK_HEME_METABOLISM
HALLMARK_GLYCOLYSIS
PROTEIN_SECRETION
HALLMARK_APOPTOSIS
Normalized Enrichment Score
E
Luminal B
HALLMARK_ESTROGEN_RESPONSE_EARLY
HALLMARK_ESTROGEN_RESPONSE_LATE
HALLMARK_TNFA_SIGNALING_VIA_NFKB
HALLMARK_HYPOXIA
HALLMARK_NOTCH_SIGNALING
HALLMARK_UV_RESPONSE_DN
HALLMARK_EPITHELIAL_MESENCHYMAL_TRANSITION
HALLMARK_APOPTOSIS
HALLMARK_GLYCOLYSIS
Normalized Enrichment Score
F
Enrichment plot: HALLMARK_ESTROGEN_RESPONSE_EARLY FDR q-value = 0.0018
Enrichment plot: HALLMARK_ESTROGEN_RESPONSE_LATE FDR q-value = 0.03
Enrichment plot: HALLMARK_TNFA_SIGNALING_VIA_NFKB FDR q-value = 0.07
Enrichment plot: HALLMARK_HYPOXIA FDR q-value = 0.14
GPx2 Low
GPx2 High
G
HER2 Enriched
HALLMARK_TNFA_SIGNALING_VIA_NFKB
HALLMARK_EPITHELIAL_MESENCHYMAL_TRANSITION
HALLMARK_MITOTIC_SPINDLE
HALLMARK_NOTCH_SIGNALING
HALLMARK_IL2_STAT5_SIGNALING
HALLMARK_INTERFERON_GAMMA_RESPONSE
HALLMARK_INFLAMMATORY_RESPONSE
HALLMARK_E2F_TARGETS
HALLMARK_APOPTOSIS
HALLMARK_HYPOXIA
HALLMARK_KRAS_SIGNALING_UP
HALLMARK_UV_RESPONSE_DN
HALLMARK_G2M_CHECKPOINT
HALLMARK_ESTROGEN_RESPONSE_EARLY
HALLMARK_ESTROGEN_RESPONSE_LATE
Normalized Enrichment Score
H
HALLMARK_EPITHELIAL_MESENCHYMAL_TRANSITION FDR q-value = 0
Enrichment plot: HALLMARK_MITOTIC_SPINDLE FDR q-value = 0.0037
Enrichment plot: HALLMARK_E2F_TARGETS FDR q-value = 0.014
Enrichment plot: HALLMARK_HYPOXIA FDR q-value = 0.015
I
Basal-like
HALLMARK_PROTEIN_SECRETION
HALLMARK_G2M_CHECKPOINT
HALLMARK_MYC_TARGETS_V1
HALLMARK_MITOTIC_SPINDLE
HALLMARK_GLYCOLYSIS
HALLMARK_UNFOLDED_PROTEIN_RESPONSE
HALLMARK_E2F_TARGETS
HALLMARK_TGF_BETA_SIGNALING
HALLMARK_DNA _REPAIR
Normalized Enrichment Score
J
Enrichment plot: HALLMARK_G2M_CHECKPOINT FDR q-value = 0.009
Enrichment plot: HALLMARK_MYC_TARGETS_V1 FDR q-value = 0.015
Enrichment plot: HALLMARK_MITOTIC_SPINDLE FDR q-value = 0.027
Enrichment plot: HALLMARK_GLYCOLYSIS FDR q-value = 0.02

Fig. S2

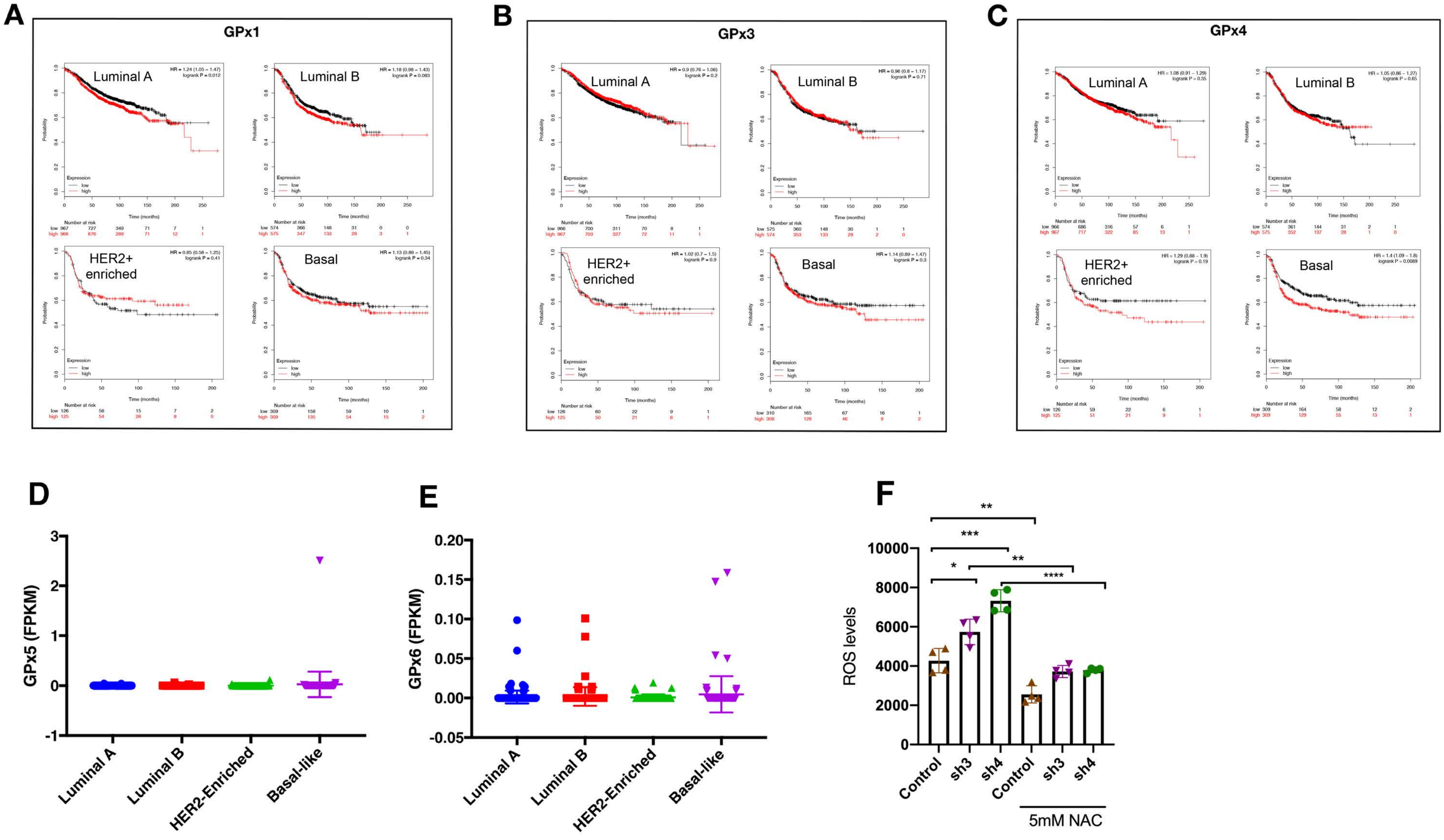

A
GPx1
Luminal A
Luminal B
HER2+ enriched
Basal
B
GPx3
C
GPx4
D
GPx5 (FPKM)
E
GPx6 (FPKM)
Luminal A
Luminal B
HER2-Enriched
Basal-like
F
ROS levels
Control
sh3
sh4
5mM NAC

Fig. S3

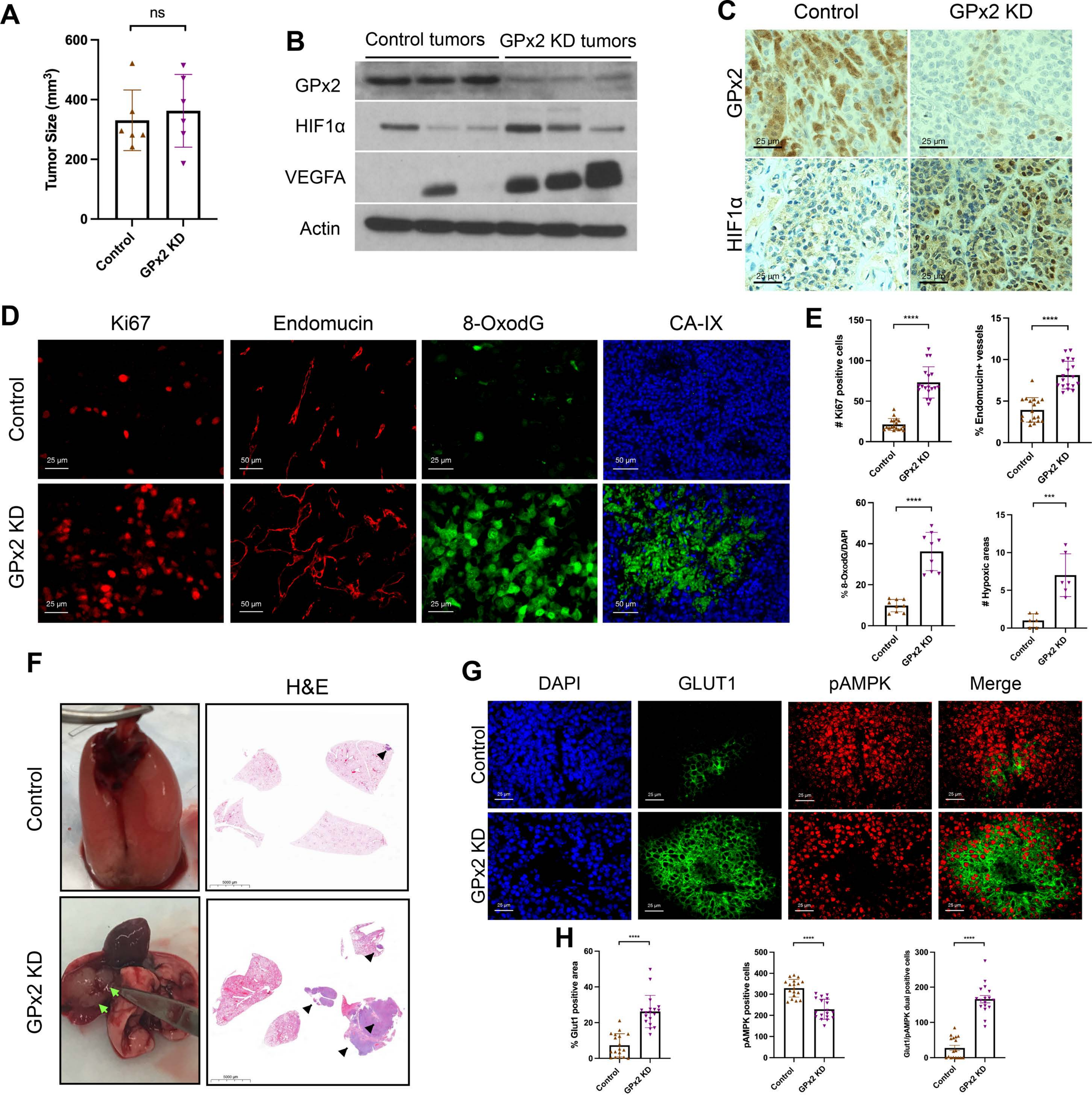
A
ns
Tumor Size (mm3)
Control
GPx2 KD
B
Control tumors
GPx2 KD tumors
GPx2
HIF1α
VEGFA
Actin
C
Control
GPx2 KD
GPx2
HIF1α
D
Ki67
Endomucin
8-OxodG
CA-IX
Control
GPx2 KD
E
Ki67 positive cells
% Endomucin+ vessels
% 8-OxodG/DAPI
# Hypoxic areas
F
H&E
Control
GPx2 KD
G
DAPI
GLUT1
pAMPK
Merge
Control
GPx2 KD
H
% Glut1 positive area
pAMPK positive cells
Glut1/pAMPK dual positive cells

Fig. S4

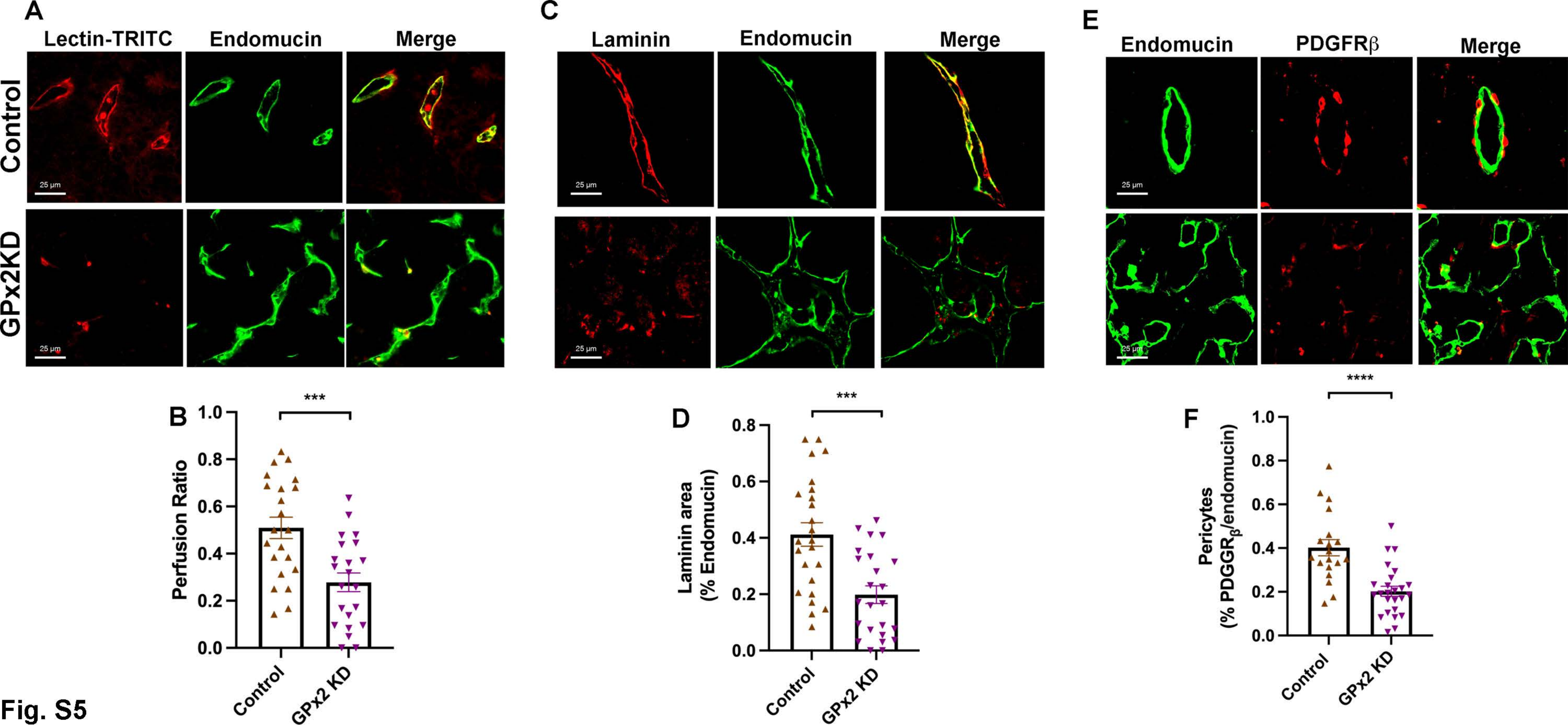
A
Lectin-TRITC
Endomucin
Merge
Control
GPx2KD
25 µm
B
Perfusion Ratio
***
Control
GPx2 KD
C
Laminin
Endomucin
Merge
D
Laminin area (% Endomucin)
***
E
Endomucin
PDGFRβ
Merge
F
Pericytes (% PDGGRβ/endomucin)
****

Fig. S5

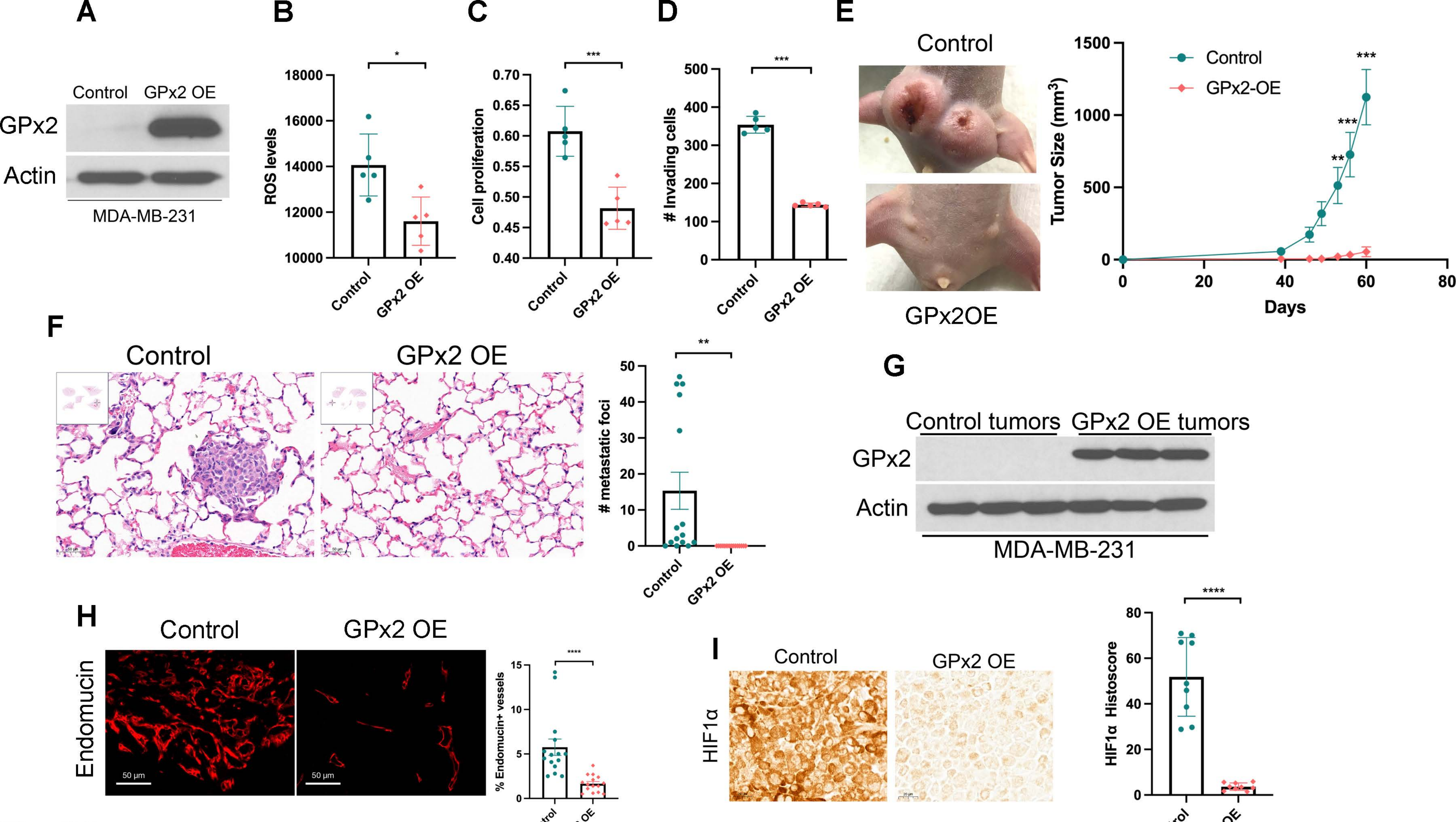
A
Control
GPx2 OE
GPx2
Actin
MDA-MB-231
B
ROS levels
Control
GPx2 OE
C
Cell proliferation
D
Invading cells
E
Control
GPx2OE
Tumor Size (mm3)
Days
GPx2-OE
F
Control
GPx2 OE
metastatic foci
G
Control tumors
GPx2 OE tumors
GPx2
Actin
MDA-MB-231
H
Control
GPx2 OE
Endomucin
50 µm
% Endomucin+ vessels
I
Control
GPx2 OE
HIF1α
HIF1α Histoscore

Fig. S6

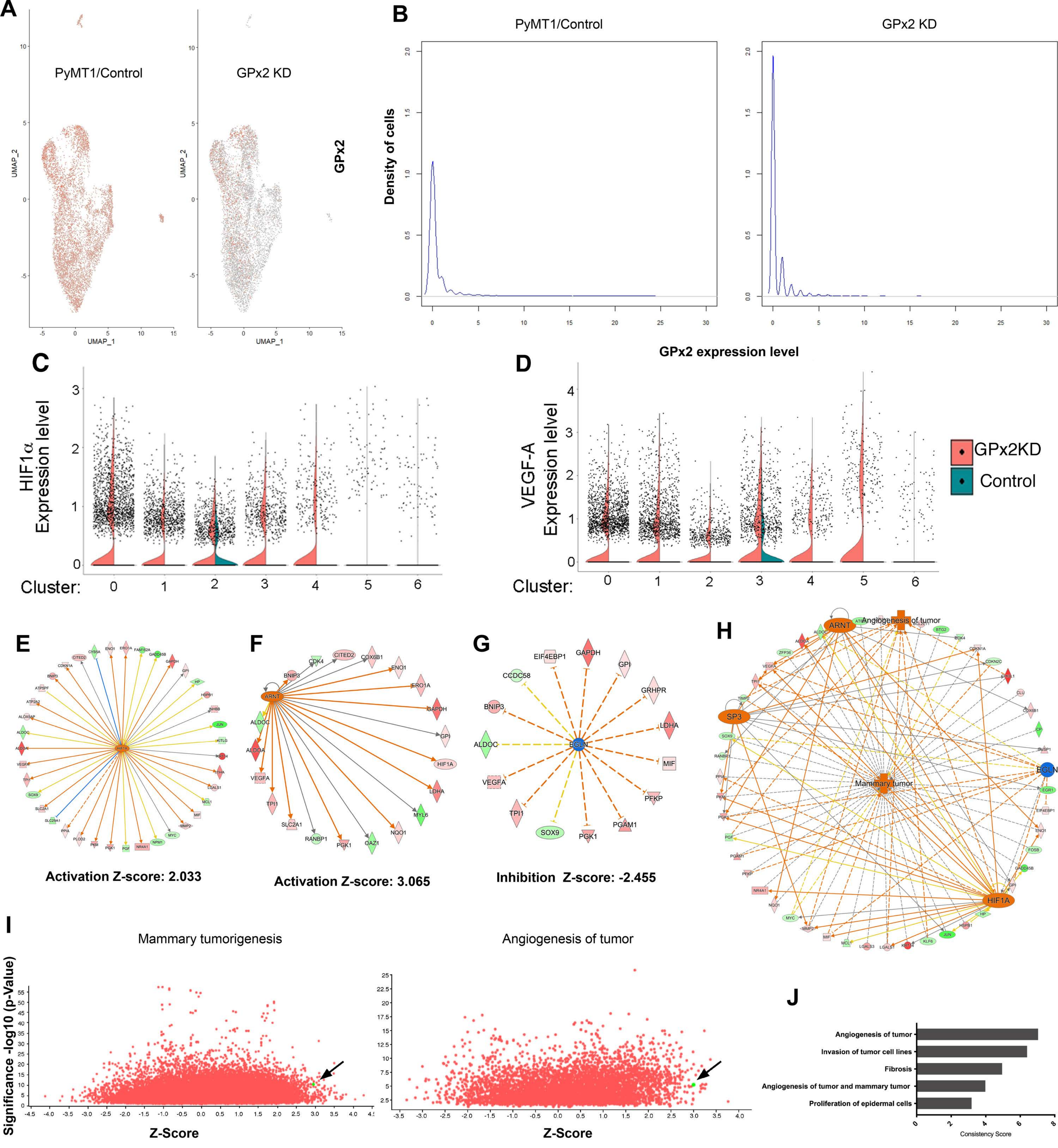

A
PyMT1/Control
GPx2 KD
GPx2
UMAP_1
UMAP_2
B
PyMT1/Control
GPx2 KD
Density of cells
C
HIF1α
Expression lelvel
Cluster:
D
GPx2 expression level
VEGF-A
Expression lelvel
Cluster:
GPx2KD
Control
E
Activation Z-score: 2.033
F
Activation Z-score: 3.065
G
Inhibition Z-score: -2.455
H
Angiogenesis of tumor
Mammary tumor
ARNT
SP3
HIF1A
EGLN
I
Mammary tumorigenesis
Angiogenesis of tumor
Significance -log10 (p-Value)
Z-Score
J
Angiogenesis of tumor
Invasion of tumor cell lines
Fibrosis
Angiogenesis of tumor and mammary tumor
Proliferation of epidermal cells
Consistency Score

Fig. S7

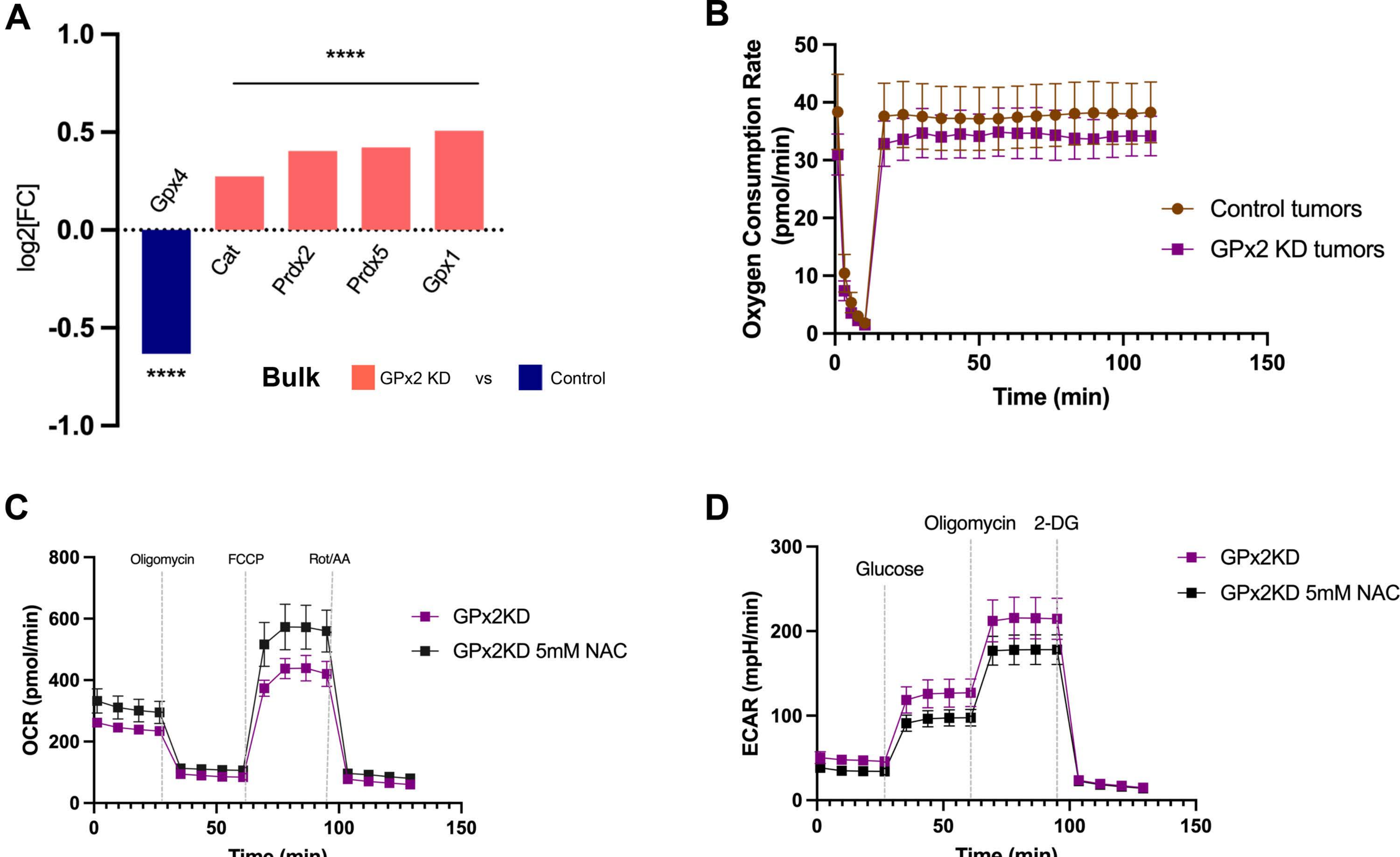
A
1.0
0.5
0.0
-0.5
-1.0
log2[FC]
****
Gpx4
Cat
Prdx2
Prdx5
Gpx1
****
Bulk
GPx2 KD
vs
Control
B
Oxygen Consumption Rate (pmol/min)
50
40
30
20
10
0
0
50
100
150
Time (min)
Control tumors
GPx2 KD tumors
C
Oligomycin
FCCP
Rot/AA
OCR (pmol/min)
800
600
400
200
0
0
50
100
150
Time (min)
GPx2KD
GPx2KD 5mM NAC
D
Glucose
Oligomycin
2-DG
ECAR (mpH/min)
300
200
100
0
0
50
100
150
Time (min)
GPx2KD
GPx2KD 5mM NAC

Fig. S8

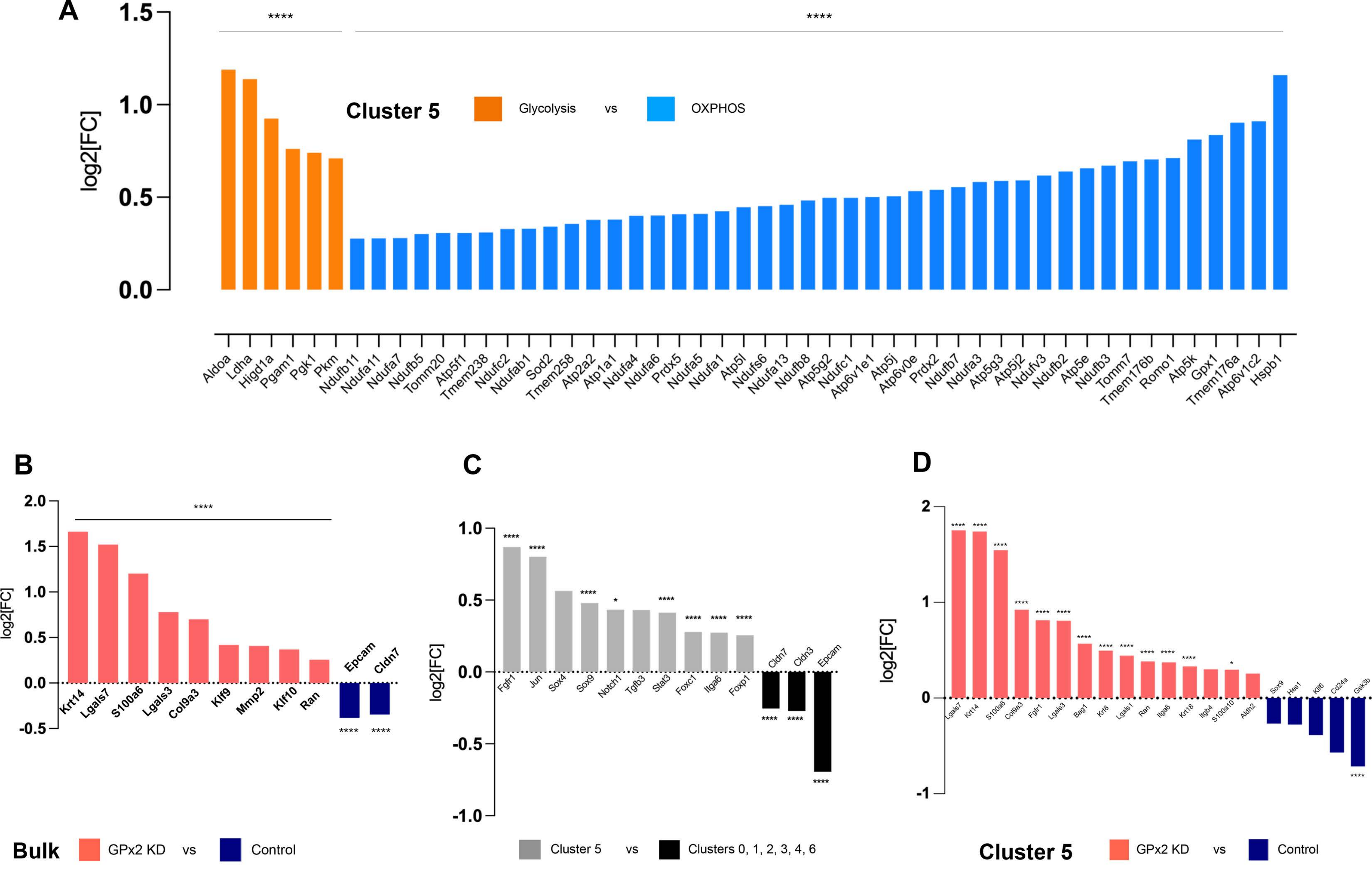
A
log2[FC]
1.5
1.0
0.5
0.0
****
****
Cluster 5
Glycolysis
vs
OXPHOS
Aldoa
Ldha
Higd1a
Pgam1
Pgk1
Pkm
Ndufb11
Ndufa11
Ndufa7
Ndufb5
Tomm20
Atp5f1
Tmem238
Ndufc2
Ndufab1
Sod2
Tmem258
Atp2a2
Atp1a1
Ndufa4
Ndufa6
Prdx5
Ndufa5
Ndufa1
Atp5l
Ndufs6
Ndufa13
Ndufb8
Atp5g2
Ndufc1
Atp6v1e1
Atp5j
Atp6v0e
Prdx2
Ndufb7
Ndufa3
Atp5g3
Atp5j2
Ndufv3
Ndufb2
Atp5e
Ndufb3
Tomm7
Tmem176b
Romo1
Atp5k
Gpx1
Tmem176a
Atp6v1c2
Hspb1
B
log2[FC]
2.0
1.5
1.0
0.5
0.0
-0.5
****
Krt14
Lgals7
S100a6
Lgals3
Col9a3
Klf9
Mmp2
Klf10
Ran
Epcam
Cldn7
Bulk
GPx2 KD
vs
Control
C
log2[FC]
1.0
0.5
0.0
-0.5
-1.0
Fgfr1
Jun
Sox4
Sox9
Notch1
Tgfb3
Stat3
Foxc1
Itga6
Foxp1
Cldn7
Cldn3
Epcam
Cluster 5
vs
Clusters 0, 1, 2, 3, 4, 6
D
log2[FC]
2
1
0
-1
Lgals7
Krt14
S100a6
Col9a3
Fgfr1
Lgals3
Bag1
Krt8
Lgals1
Ran
Itga6
Krt18
Itgb4
S100a10
Aldh2
Sox9
Hes1
Klf6
Cd24a
Gsk3b
Cluster 5
GPx2 KD
vs
Control

Fig. S9

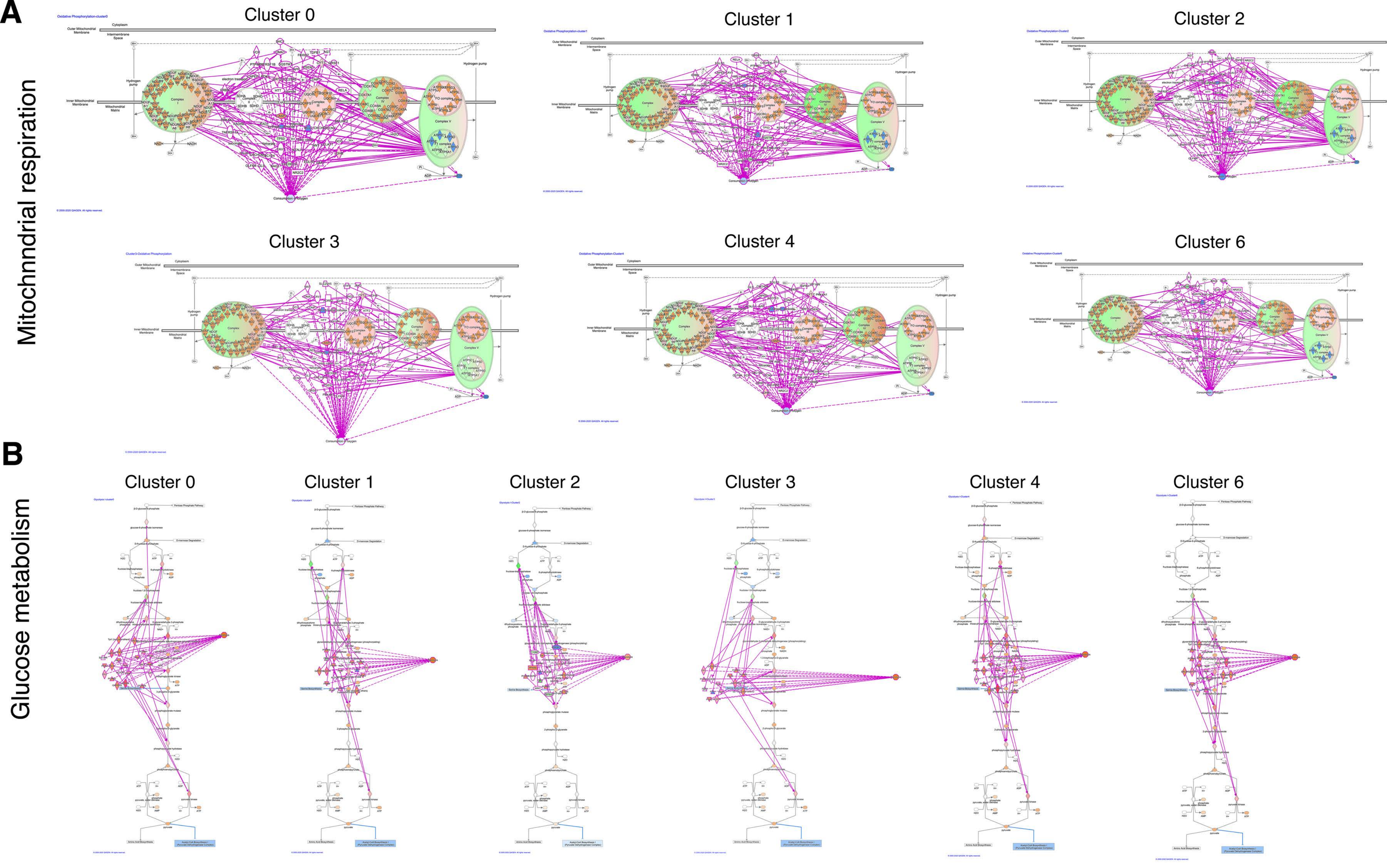
A
Mitochnndrial respiration
Cluster 0
Cluster 1
Cluster 2
Cluster 3
Cluster 4
Cluster 6
B
Glucose metabolism
Cluster 0
Cluster 1
Cluster 2
Cluster 3
Cluster 4
Cluster 6

Fig. S10

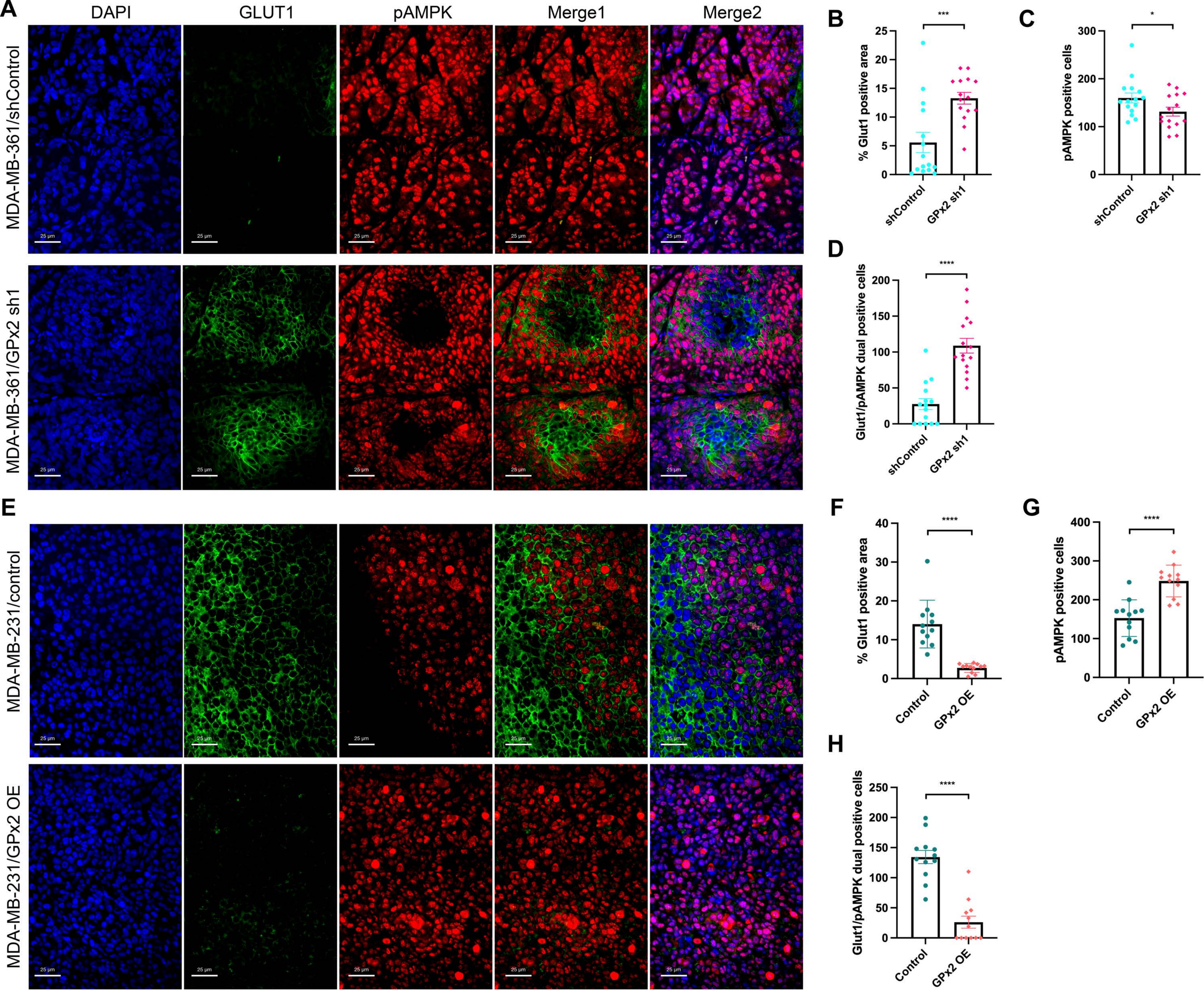
A
DAPI
GLUT1
pAMPK
Merge1
Merge2
MDA-MB-361/shControl
MDA-MB-361/GPx2 sh1
25 µm
B
% Glut1 positive area
***
shControl
GPx2 sh1
C
pAMPK positive cells
*
shControl
GPx2 sh1
D
Glut1/pAMPK dual positive cells
****
shControl
GPx2 sh1
E
MDA-MB-231/control
MDA-MB-231/GPx2 OE
F
% Glut1 positive area
****
Control
GPx2 OE
G
pAMPK positive cells
****
Control
GPx2 OE
H
Glut1/pAMPK dual positive cells
****
Control
GPx2 OE

Fig. S11

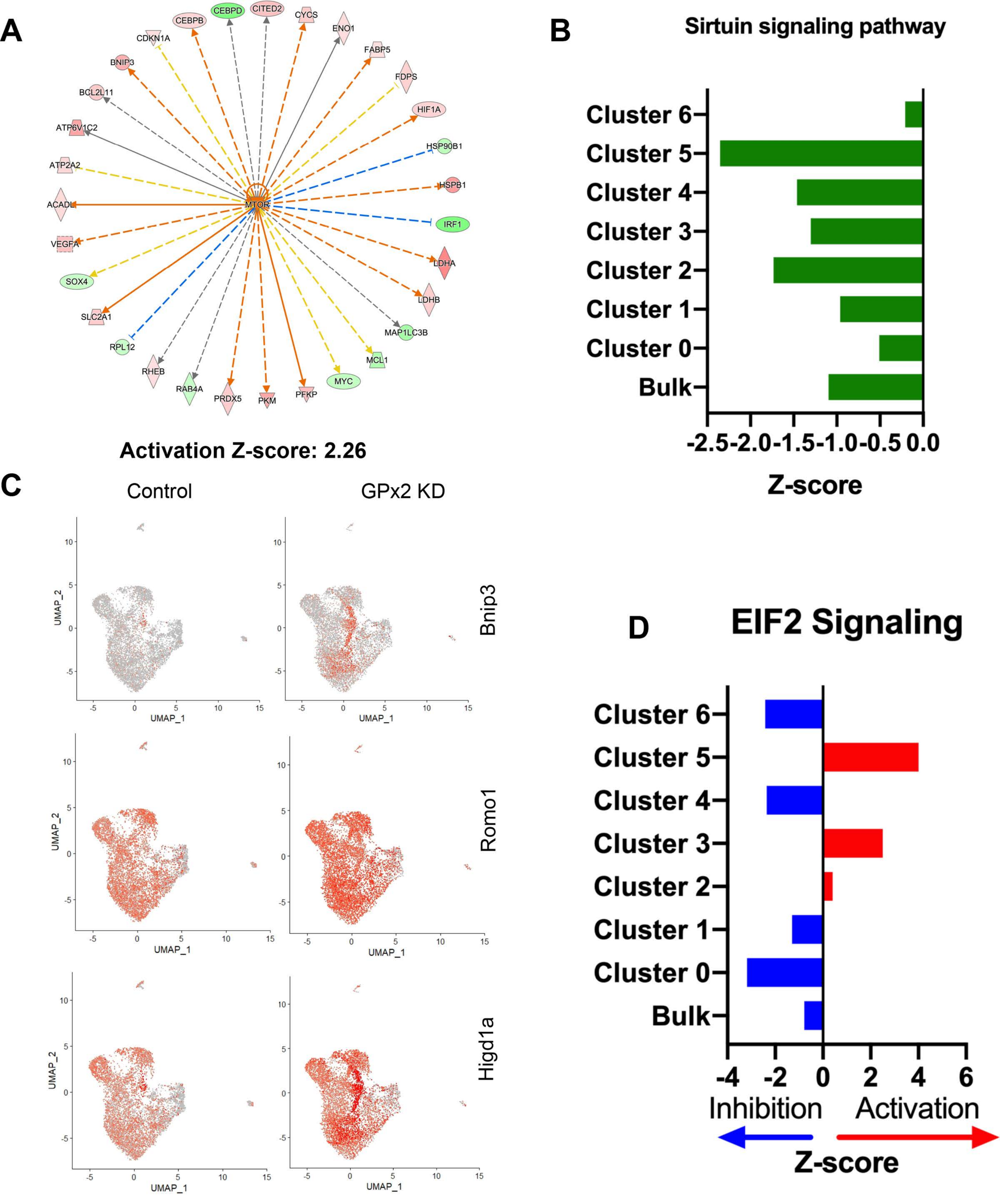
A
CEBPD
CITED2
CEBPB
CYCS
ENO1
CDKN1A
FABP5
BNIP3
FDPS
BCL2L11
HIF1A
ATP6V1C2
HSP90B1
ATP2A2
HSPB1
MTOR
ACADL
IRF1
VEGFA
LDHA
SOX4
LDHB
SLC2A1
MAP1LC3B
RPL12
MCL1
RHEB
MYC
RAB4A
PRDX5
PKM
PFKP
Activation Z-score: 2.26
B
Sirtuin signaling pathway
Cluster 6
Cluster 5
Cluster 4
Cluster 3
Cluster 2
Cluster 1
Cluster 0
Bulk
-2.5-2.0-1.5-1.0-0.5 0.0
Z-score
C
Control
GPx2 KD
Bnip3
Romo1
Higd1a
UMAP_1
UMAP_2
D
EIF2 Signaling
Cluster 6
Cluster 5
Cluster 4
Cluster 3
Cluster 2
Cluster 1
Cluster 0
Bulk
-4 -2 0 2 4 6
Inhibition
Activation
Z-score

Fig. S12

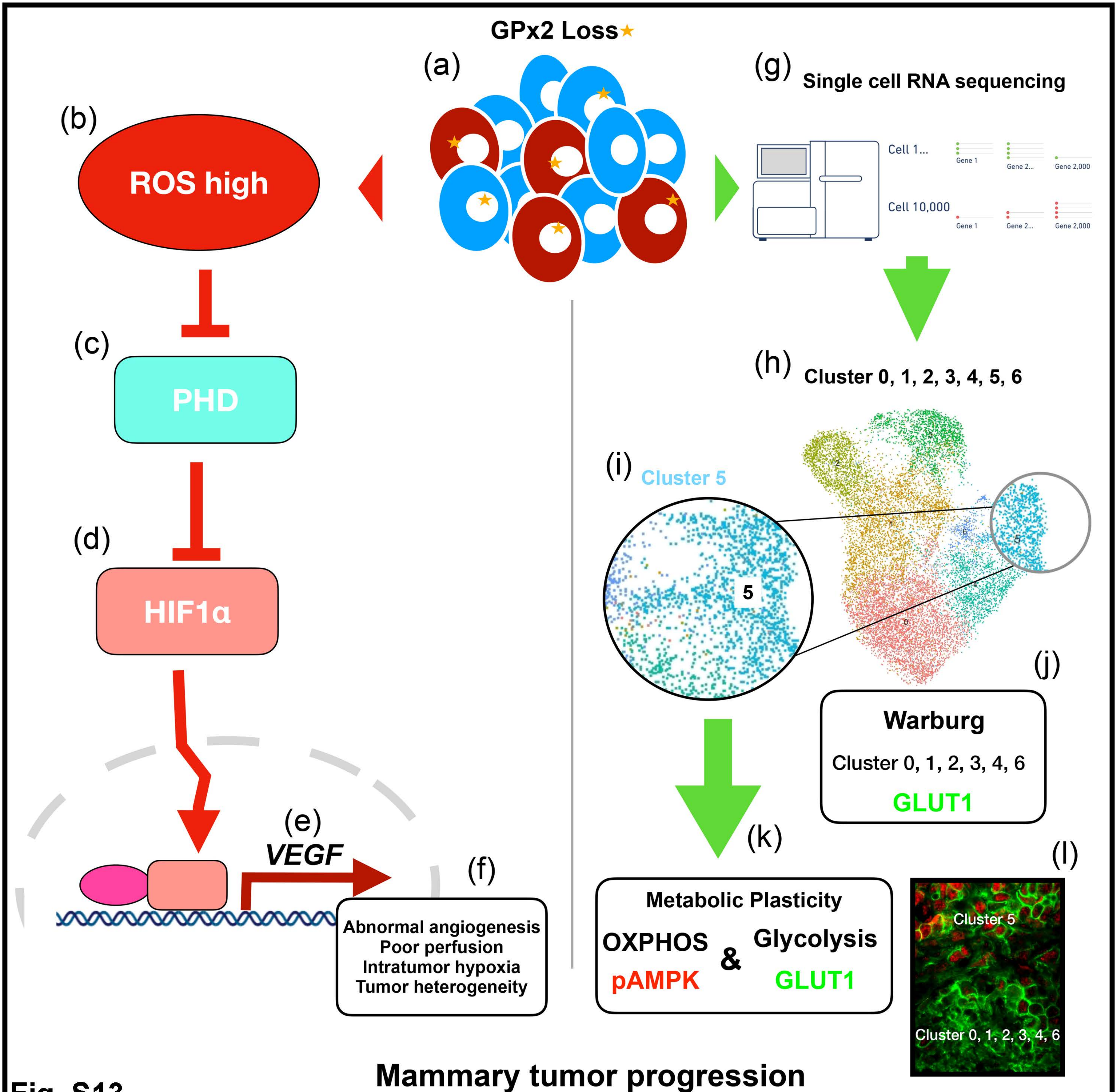
GPx2 Loss
(a)
(b)
ROS high
(c)
PHD
(d)
HIF1α
(e)
VEGF
(f)
Abnormal angiogenesis
Poor perfusion
Intratumor hypoxia
Tumor heterogeneity
(g)
Single cell RNA sequencing
Cell 1...
Cell 10,000
Gene 1
Gene 2...
Gene 2,000
(h)
Cluster 0, 1, 2, 3, 4, 5, 6
(i)
Cluster 5
5
(j)
Warburg
Cluster 0, 1, 2, 3, 4, 6
GLUT1
(k)
Metabolic Plasticity
OXPHOS
pAMPK
&
Glycolysis
GLUT1
(l)
Cluster 5
Cluster 0, 1, 2, 3, 4, 6
Mammary tumor progression

Fig. S13

Supplemental Figure legends

Fig. S1. GPx2 KD in PyMT1 mammary tumor cells stimulates malignant traits. (**A**) PyMT1 and PyMT2 cell lines ($1x10^6$) were bilaterally injected into mammary glands of female athymic nude mice (n=3). Representative images of lung metastases at 40 days post tumor onset are shown. (**B-C**) The number and diameter of metastases generated by PyMT2 relative to PyMT1 cells is displayed as Mean ± SEM; ** $p < 0.01$. (**D**) GPx2 expression in PyMT1 and PyMT2 cell lines was tested by immunoblotting with anti-GPx2 antibody. (**E**) ROS levels in PyMT1 and PyMT2 cells were tested by H2DCFDA fluorescence using a DCF probe; Mean ± SEM; **** $p < 0.0001$. (**F**) GPx2 immunoblots of PyMT1 cells expressing control shRNA or 5 GPx2 shRNAs cells. ROS levels in PyMT1 control and PyMT1 cells expressing sh3, sh4, and sh5 were untreated or treated with 1uM H2O2 and shown as Mean±SEM; * $p < 0.05$; ** $p < 0.01$, *** $p < 0.001$, **** $p < 0.0001$. (**G**) Cell proliferation of PyMT1/GPx2 KD (sh3 and sh4) vs PyMT1 control cells was measured 48 hrs post plating by the WST-1 assay; Mean ± SEM; ** $p < 0.01$; *** $p < 0.001$. (**H**) PyMT1/GPx2 KD and PyMT1 control cells were compared for spheroid formation in Mean ± SEM; *** $p < 0.001$, or (**I**) Matrigel invasion; Mean ± SEM; ** $p < 0.01$, *** $p<0.001$. Triplicates were used.

Fig. S2. GPx2 under-expression in breast cancer subtypes is associated with oncogenic signaling and poor patient survival. (**A**) TCGA mining of RNAseq dataset on human breast tumors and matched normal breast tissue. The level and distribution of GPx2 mRNA in normal versus cancer is shown as Mean ± SEM; ****$p < 0.0001$. (**B**) TCGA breast cancer dataset analysis for GPx2 mRNA expression in four breast cancer subtypes showed a significance difference between luminal B, HER2+, and Basal-like relative to luminal A tumors ($p<0.05$), but insignificant difference between luminal B and luminal A ($p>0.05$). (**C**) Kaplan-Meier analysis showed a significant association between low GPx2 mRNA in luminal B, HER2-enriched and Basal-like subtypes with shorter survival duration ($p<0.05$), but not luminal A tumors ($p=0.82$). (**D-J**) Gene set enrichment analysis (GSEA) of breast cancer subtypes show that luminal A tumors with low GPx2 expression were not significantly enriched in oncogenic signaling except for a trend for metabolic pathways (D), whereas

low GPx2 expressing luminal B (E-F), HER2+ (G-H) and Basal-like tumors (I-J) were enriched in the indicated oncogenic pathways relative to tumors with high GPx2 mRNA expression.

Fig. S3. GPx1, GPx3, and GPx4 mRNA relationship with patient survival. (**A-B**) GPx1 and GPx3 mRNA expression in luminal A, luminal B, HER2-enriched, or Basal-like breast tumors was not significantly associated with patient survival; ns=p>0.05. (**C**) High GPx4 expression in Basal-like cancers was correlated with shorter patient survival duration; $p<0.05$. (**D-E**) Analysis of TCGA breast cancer dataset on GPx5 and GPx6 mRNA in luminal A, luminal B, HER2-enriched, and Basal-like subtypes did not show significant expression. (**F**) ROS levels in PyMT1 control and PyMT1/GPx2 sh3 and sh4 cells (4 replicas) were tested in cells treated -/+ 5mM NAC; Mean ± SEM; * $p < 0.05$; ** $p < 0.01$, *** $p < 0.001$, **** $p < 0.0001$.

Fig. S4. **GPx2 KD promotes cell proliferation, angiogenesis, oxidative stress, hypoxia, and metastasis, independently of tumor bulkiness.** (**A**) Similarly-sized tumors (n=6) from 3 mice injected with PyMT1 control and PyMT1/GPx2 KD cells ($1X10^6$) were removed after tumor growth to 10 mm in diameter (34 days for control and 27 days for GPx2 KD). Bar graphs display tumor volume at end point; Mean ± SEM; ns=p>0.05. (**B**) Same size control and GPx2 KD tumors (n=3 from 3 independent mice) were immunoblotted with anti GPx2, HIF1α, VEGFA, or Actin antibody. (**C**) Representative images of GPx2 and HIF1α IHC staining of similarly sized control and GPx2 KD tumors (3 sections each) are shown. (**D**) Three random sections from control and GPx2 KD tumors were immunostained for Ki67, endomucin, 8-OxodG, and CA-IX. (**E**) Bar graphs display per section the number of Ki67 positive tumor cells, the fraction of endomucin+ vessels, the percentage of 8-OxodG positive cells per section, and the number of CA-IX positive areas; Mean ± SEM; ***$p< 0.001$, ****$p < 0.0001$. the fraction of endomucin labeled vessels per section, the percentage of 8-OxodG positive cells, and the number of CA-IX positive areas; Mean ± SEM; ***$p< 0.001$, ****$p < 0.0001$. (**F**) Mice bearing same size control and GPx2 KD tumors underwent surgery to remove primary tumor; 35 days post surgery, lung metastasis was examined. Scans of whole lung lobes (boxes) from H&E stained lung sections from mice control and GPx2 KD tumors are shown. (**G**) Similarly-sized tumors (n=6) of each group were sectioned and 3 random sections from each tumor were co-immunostained with anti GLUT1 (FITC) and p-AMPK (TRITC) antibody and counterstained with DAPI (blue). (**H**) The fraction of GLUT1 positive tumor area per section was

quantified by ImageJ (left bar graphs); Mean ±SEM; the number of p-AMPK positive tumor cells (middle bar graphs) and the number of GLUT1/p-AMPK dual positive tumor cells (right bar graphs) per section were calculated as Mean ± SEM. Two-tailed t-test; ****$p < 0.0001$.

Fig. S5. **GPx2 KD results in vascular malfunction due to poor perfusion linked to impaired vascular integrity and pericyte coverage.** (**A**) Mice bearing PyMT1 control and PyMT1/GPx2 KD tumors (3 mice each) were injected intravenously with 50µg DyLight® 594 tomato lectin (TRITC); tumors were fixed and two random sections per tumor were immunostained with anti-endomucin (FITC). (**B**) Perfusion ratio or the percentage of Lectin-TRITC positive vessels co-localizing with endomucin positive vessels was analyzed by imageJ; Mean ± SEM; ***$p < 0.001$. (**C**) PyMT1 control and PyMT1/GPx2 KD tumors (5 mice each) were co-immunostained with anti-endomucin (FITC) and anti-laminin (TRITC). Endomucin/laminin co-localized vessels are mature vessels. (**D**) The maturation fraction or the ratio of laminin positive vessels over endomucin labeled vessels was analyzed by imageJ; Mean ± SEM; ***$p < 0.001$. (**E**) PyMT1 control and PyMT1/GPx2 KD tumors (5 mice each) were co-stained with anti-endomucin (FITC) and anti-PDGFRβ (TRITC) to label vessels and pericytes respectively; (**F**) the percentage of PDGFRβ/endomucin co-localized vessels is shown as Mean ± SEM; ****$p < 0.0001$.

Fig. S6. **GPx2 overexpression in MDA-MB-231 cells inhibits ROS, vascular density, hypoxia and malignant traits.** (**A**) MDA-MB-231 cells expressing control vector or human GPx2 lentiviral vector were immunoblotted for GPx2 or Actin ; or tested for (**B**) ROS levels; Mean ± SEM; *$p < 0.05$; **C**) 24 hr cell proliferation using WST-1 assay; Mean ± SEM; ***$p < 0.001$; (**D**) Matrigel invasion; Mean ± SEM; ***$p < 0.001$. (**E**) Mammary tumor growth curves of mice (n=3 each) injected with MDA-MB-231 control or GPx2 OE cells ($1x10^6$) are shown as tumor volume; Mean ± SEM; **$p < 0.01$, ***$p < 0.001$; representative images of tumor growth at 60 days post onset are shown. (**F**) H&E stained lung sections from scans of whole lung lobes (boxes) from mice carrying control and GPx2 OE tumors; The number and distribution of metastatic foci generated by MDA-MB-231 control and GPx2 OE tumors is shown as Mean ±SEM; **$p < 0.01$. (**G**) MDA-MB-231 control and GPx2 OE tumors (n=3) were immunoblotted for GPx2 and Actin or (**H**) immunostained for endomucin (FITC) (n=6 tumors); the fraction of endomucin+ vessels per section (3 sections each) was quantified by

ImageJ; Mean ± SEM; ****$p < 0.0001$; or (**I**) immunostained for HIF1α and quantified by histoscore; Mean ± SEM; ****$p < 0.0001$.

Fig. S7. **scRNA-seq based core analysis by IPA reveals GPx2 KD-stimulated increases in HIF1α and VEGFA signaling.** (**A**) Feature plots in low-dimensional space illustrate GPx2 mRNA feature expression in PyMT1 control versus PyMT1/GPx2 KD tumor. (**B**) GPx2 expression levels in control and GPx2 KD tumor; X-axis indicates GPx2 mRNA expression level; Y-axis indicates density of cells expressing GPx2 mRNA. PyMT1/GPx2 KD tumor cells are peaked in zero GPx2 expression relative to PyMT1 control cells. (**C-D**) Violin plots visualize single cell expression distributions of HIF1α and VEGFA mRNA expression in tumor cell clusters 0 to 6 in GPx2 KD tumor (red) relative to the control tumor clusters (green). (**E-G**) IPA upstream regulator analysis predicts that GPx2 KD activates (E) HIF1α (Z-score=2.033) and (F) ARNT (HIF1β) (Z-score=3.065), while (G) inhibiting EGLN (PHD) (Z-score= -2.455). Z-score > 2 or < -2 indicates activation or inhibition, respectively. (**H, I**) IPA regulator effect analysis revealed (H) significant disease and function model based on differentially expressed genes, (I) with Z-score ~3.0, indicating activation. (J) Both models were top-ranked ones upon consistency

Fig. S8. **GPx2 loss upregulates other antioxidants and inhibits *OCR in vivo and mediates* ROS effects on metabolism.** (**A**) Bar graph showing $\log_2$[FC] values for the differentially expressed genes in the GPx2 KD tumor cells (n=7725) relative to control tumor cells (n=7374) for other members of antioxidant enzymes. The Log transformation is at base 2 of mRNA fold change; a log(FC) of 0 means no difference; a log(FC) less or greater than 0 means downregulated or upregulated mRNA expression respectively; ****$p < 0.0001$. (**B**) PyMT1/GPx2 KD tumors were compared to control PyMT1 tumors (n=8) *ex vivo* for OCR. (**C-D**) PyMT1/GPx2 KD cells that were treated or untreated with 5mM NAC were assayed for OCR (C) and ECAR (D).

Fig. S9. Tumor cells subpopulations in cluster 5 display gene signatures associated with EMT and stemness. (**A**) Bar graph displays the differentially expressed OXPHOS- and glycolysis-associated genes in cluster 5 comparing GPx2 KD tumor cells (n=520) and control tumor cells (720); ****$p < 0.0001$. (**B**) Bar graphs display $\log_2$[FC] values for the differentially expressed genes-associated with EMT/stemness in the GPx2 KD tumor cells (n=7725) relative to control tumor cells (n=7374); ****$p < 0.0001$. (**C**) Bar graph showing $\log_2$[FC]

values for the differentially expressed genes-associated with EMT and stemness in cluster 5 (cell number: n=1235) relative to all other clusters (0, 1, 2, 3, 4, 6) (total cell number: n=13864); *p < 0.05 ; ****p < 0.0001.

(**D**) Bar graph showing $\log_2$[FC] values for the differentially expressed genes-associated with EMT/stemness in cluster 5 comparing GPx2 KD tumor cells (n=520) and control tumor cells (720); *p < 0.05 ; ****p < 0.0001.

Fig. S10. GPx2 knockdown inhibits oxygen consumption and activates glycolysis in all tumor clusters except for cluster 5. (**A-B**) Overlay of the differentially expressed genes by GPx2 KD in clusters 0, 1, 2, 3, 4, 6 onto OXPHOS and glycolysis pathways individually in Ingenuity Knowledge Base predicts inhibition (blue hexagon) and activation (orange hexagon) of oxygen consumption and glycolysis, respectively.

Fig. S11. GPx2 loss stimulates a hybrid metabolic phenotype in human breast cancer cells. (**A**,**E**) MDA-MB-361 control and GPx2 sh1 mammary tumors (n=6) from 3 mice each, MDA-MB-231 control and GPx2OE tumors (n=6) derived from 3 mice each, were sectioned and three random sections from each tumor were co-immunostained with anti GLUT1 (FITC) and p-AMPK (TRITC) antibody and counterstained with DAPI (blue). (**B, F**) The fraction of GLUT1 positive tumor area per section was quantified by ImageJ; Mean ± SEM; (**C, G**) the number of p-AMPK positive tumor cells and (**D, H**) the number of GLUT1/p-AMPK dual positive tumor cells per section were calculated as Mean ± SEM. Two-tailed t-test; *p<0.05, ***p< 0.001, ****p < 0.0001.

Fig. S12. Genes and pathways were affected by GPx2 KD. (**A**) IPA upstream regulator analysis predicts that GPx2 KD activates mTOR kinase (Z-score = 2.26); Z-score > 2 or < -2 indicates activation or inhibition, respectively. (**B**) Z-score across bulk cell populations and individual clusters (0, 1, 2, 3, 4, 5, 6) for Sirtuin signaling pathway affected by GPx2 KD. (**C**) Feature plots in low-dimensional space showing increased expression of Bnip3, Romo1, and Higd1a in the GPx2 KD relative to control tumor. (**D**) Z-score across bulk cell populations and individual clusters for EIF2 signaling pathway affected by GPx2 KD.

Fig. S12. A link between redox signaling, tumor heterogeneity, metabolic plasticity, and malignant progression. GPx2 loss in mammary tumor cells (**a**) increases ROS production (**b**), which inhibits Propyl hydroxylase D (PHD) protein (**c**), thereby stabilizing HIF1α (**d**), resulting in VEGFA upregulation (**e**), causing abnormal angiogenesis, due to a non-perfusing vasculature, resulting in hypoxia and tumor heterogeneity (**f**). Single cell RNA sequencing of the GPx2 KD tumor (**g**) revealed 7 epithelial tumor cell clusters (**h**), which, with

the exception of cluster 5 (**i**), exhibited aerobic glycolysis or Warburg effect (**j**). By contrast, cluster 5 was endowed with the ability to use both OXPHOS and glycolysis, implying metabolic plasticity (**k**). The latter was demonstrated by the dual expression of p-AMPK (TRITC) and GLUT1 (FITC) in cluster 5-like cells, as markers of OXPHOS and glycolysis respectively (**l**). By contrast, clusters 0, 1, 2, 3, 4, 6, which exhibit the Warburg effect, are predominantly GLUT1 (FITC) positive (**l**). These data suggest that cluster 5 may drive tumor cell adaptation and survival, underlying an aggressive phenotype leading to tumor progression.

Supplemental Methods

Cell lines

Primary mammary tumor cell lines were prepared as follows. Tumors were excised, washed in PBS and diced with a razor blade into smaller fragments. Each gram of tumor was incubated with 10 mL of digestion media (1 mg/mL Collagenase type I, 100 unit/ml Hyaluronidase, 100 units/ml Penicillin/ streptomycin, 2 mg/ml of BSA in Medium 199). The suspension was incubated at 37 ºC for 3 hrs with occasional mixing. Digested material was spun at 1000 rpm for 5 min and pelleted cells were plated overnight at high density (1X107 cells per 10 cm dish) in DME/10%FBS supplemented with 10 µg/ml insulin and 20 µg/ml EGF. Fibroblasts were depleted from epithelial tumor cells by limiting trypsinization. Epithelial cells were grown in culture for several weeks till they reached crisis and adapted to in vitro culture conditions. The human MDA-MB-361 and MDA-MB-231 breast cancer cell lines were cultured in DMEM medium (Gibco) supplemented with 10% FBS and 1% Pen-Strep (Gibco).

Animal studies

Animal protocols used for this study were reviewed and approved by the Institute for Animal Studies at the Albert Einstein College of Medicine. Mice were housed and maintained by the Animal Studies Institute at the Albert Einstein College of Medicine. Animal protocols used for this study were reviewed and approved by the Institute for Animal Studies. The mice were obtained from Jackson laboratories. MMTV-PyMT transgenic mice that were used in another study were derived by brother-sister (sibling) mating of progeny from a cross of p21 knockout female (Bl6/129S/ background) with MMTV-PyMT heterozygous male (FVB background) (1). Only female p21wt/wt PyMT mice derived from F2 progeny of selected brother sister matings were used to

derive primary mammary tumor cell lines. These resulting recombinant mice were on a mixed Bl6/129S/ FVB/N background that were backcrossed in the FVB/N background. PyMT xenograft models were generated by injection of p21wt/wt PyMT cell lines derived from the same mammary tumor (PyMT1 and PyMT2) into female athymic nude mice obtained from Charles River. For MDA-MB-361 xenografts, we subcutaneously implanted estrogen pellets (0.36mg 17 beta-estradiol pellet, 90 day release, Innovative Research of America) into athymic female nude mice and injected 4 million tumor cells (suspended in PBS:Matrigel 1:1) into two mammary fat pads of nude mice 24 hours later. For MDA-MB-231 xenografts, 1 million cells suspended in 200 ul PBS containing 25% Matrigel were bilaterally injected in the flanks of athymic female nude mice.

Tumor growth kinetics

Tumor growth curve was determined by measuring tumor size. The tumor size was measured twice a week using a caliper. Tumor volume was determined upon the formula: tumor volume = shorter diameter2 X longer diameter/2. Mice (five per group for PyMT1 vs. PyMT1/GPx2 KD; four for PyMT2 vs five for PyMT2/GPx2-OE; three for MDA-MD-361 vs three for MDA-MB-361/GPx2sh1) were sacrificed at end point. Data are displayed as mean tumor volume -/+ SEM. Statistical analyses were performed comparing individual time points by unpaired t-test and significant differences were established as p-value < 0.05.

Lung metastasis

Mice bearing control and GPx2 KD or GPx2 OE mammary tumors were sacrificed at end point of 1-2 cm diameter, as allowed by our animal protocol. Lungs were inflated by tracheal cannulation with injection of 1-2 ml of 10% neutral buffered formalin. Formalin fixed lungs were paraffin embedded and blocks sectioned on a tissue microtome (Leica Microsystems) at 5 µm. Lungs were serial sectioned through the tissue and sets of 5 serial sections, at 300 um intervals. Analysis was performed on whole sections after it was determined by inspection that metastases seeded in random locations. Data are displayed as mean foci number ± SEM. Statistical analyses were performed using unpaired t-test and significance determined at $p < 0.05$.

Constructs

Mouse GPx2 plasmid (GenScript) was subcloned by PCR into Xho1/BamH1 restriction sites of lentiviral expression vector pLVX-puro (Clontech), using 5' region primer: 5'-TAT CTC GAG GCC ACC ATG GCT TAC ATT GCC AAG TCG-3' and 3' region primer: 5'-TAT GGA TCC CTA GAT GGC AAC TTT GAG GAG CCG-3'.

Lentiviral particles produced from the obtained mGPx2-pLVX-puro plasmid were used for expression of mGPx2 protein in the PyMT2 mammary tumor cell line.

Lentivirus production

Lentiviral particles were generated by transient co-transfection of 293T cells with lentivirus-based vector expressing either the full length clone of desired gene or an shRNA sequence to target-specific RNA of gene. Briefly, a 100-mm dish seeded with 3X106 cells were transfected with 0.6 µg of lentiviral packaging gene TAT, 102 RVE, and GAG/POL and 1.2 µg of VSV-G and 12 µg of DNA of interest in lentiviral backbone. Fugene was used to transfect the cells. Following 48 hours of transfection, supernatant was collected, centrifuged at 2,000 rpm for 10 minutes, and filter sterilized

ShRNA lentiviral transduction

Lentiviral mission shRNA clones against mouse GPx2 (TRCN0000076528, TRCN0000076529, TRCN0000076530, TRCN0000076531, and TRCN0000076532) or human GPx2 (TRCN0000046241 and TRCN0000046242) were purchased from Sigma. ShRNA lentiviral vectors against mouse or human GPx2 and a control non-targeting shRNA were packaged in 293T cells and infected into cells. Briefly, PyMT1 or MDA-MB-361 cells were seeded at of 1X105 per 12-well plate for 24 hrs, treated with 250 µl viral solution containing 10 µg/ml polybrene for 1 hour and incubated in DMEM/10 % FBS without antibiotics for 24 hours. Cells were expanded into a 10-cm dish with selective antibiotics.

GPx2 lentivirus overexpression

The open reading frame of mouse GPx2 (GenScript NM_030677.2) was subcloned by PCR into Xho1/BamH1 restriction sites of lentiviral expression vector pLVX-puro (Clontech), using 5' region primer: 5'-TAT CTC GAG GCC ACC ATG GCT TAC ATT GCC AAG TCG-3' and 3' region primer: 5'-TAT GGA TCC CTA GAT GGC AAC TTT GAG GAG CCG-3’, and the open reading frame of human GPx2 (GenScript NM_002083.4) was subcloned by PCR into Xho1/BamH1 restriction sites of lentiviral expression vector pLVX-puro (Clontech), using 5’ region primer: 5’-CCC CTC GAG ATG GCT TTC ATT GCC AAG TCC TTC TAT GAC-3’ and 3' region primer: 5’-CCC GGA TCC CTA TAT GGC AAC TTT AAG GAG GCG CTT GAT-3’. Recombinant lentiviruses expressing mGPx2 and hGPx2 were packaged in 293T cells and expressed in PyMT2 cells as described above.

Real time qRT-PCR

RNA was isolated using an RNeasy Mini Kit (QIAGEN) following the manufacturer's protocol. RNA-to-CT 1-Step Kit and TaqMan primer pairs for mouse VEGFA (Mm00437306_m1) and mouse GAPDH (Mm99999915_g1), were obtained from Applied Biosystems. Real-time CR experiments were performed according to the manufacturer's protocol using a StepOnePlus Real-Time PCR Instrument from Applied Biosystems. Murine GAPDH was used as internal reference gene and relative mRNA levels of candidate genes were determined using the 2(-ΔΔCT) method. Three independent experiments were performed. Statistical significance was calculated using unpaired t-test, $p < 0.05$.

Antibodies

Antibodies against GPx2, Ki67, 8-OHdG, HIF1α, p-AMPK were obtained from Abcam. Mouse monoclonal anti VEGFA, CA-IX, ALDOA, rat monoclonal anti-Endomucin, and goat polyclonal anti-Laminin were from Santa Cruz Biotechnology. Rabbit anti-SLC2A1(GLUT1) antibody was purchased from BioSource. Mouse monoclonal anti-SLC2A1(GLUT1) antibody was from Novus-Biologicals. β-actin antibody was from Sigma. HRP conjugated anti-mouse and anti-rabbit secondary antibodies were from Jackson Laboratories. FITC or TRITC coupled secondary antibodies (Alexa Fluor 594 goat anti-rabbit, Alexa Fluor 488 goat anti-mouse, Alexa Fluor 594 donkey anti-rat, Alexa Fluor 488 donkey anti-rat, Alexa Fluor 594 donkey anti-goat) were from Invitrogen. Dako Envision Labeled Polymer-HRP anti-mouse or anti-rabbit secondary antibodies was from DAKO.

Immunoblotting

Cells or tissues were extracted in RIPA solubilization buffer (50 mM Tris-HCL pH 7.5), 150 mM NaCl, 0.5mM $MgCl_2$, 0.2mM EGTA, 1% Triton X-100) including protease and phosphatase inhibitors. 30 µg protein were loaded on 7-12% SDS-polyacrylamide gels and transferred to Immobilon membranes. Blots were probed overnight at 4 ºC with indicated antibodies and developed by chemiluminescence (Perkin Elmer).

Immunofluorescence

Cells seeded on coverslips were fixed in 3.7% paraformaldehyde in PBS, permeabilized in 0.01% Triton X-100, washed in PBS and blocked in 2% BSA in PBS for 1hr. Cells were incubated with primary antibody in

PBS/0.5% BSA for 2 hrs, washed and incubated with 1:2000 Alexa Fluor secondary antibody for 1 hr. Cells were washed in PBS/0.5% BSA and counterstained with DAPI. For tissue immunofluorescence, formalin-fixed/paraffin-embedded tumor tissues were sectioned in 5 μm thickness, deparaffinized in xylene, and rehydrated in a series of 100% ethanol, 95% ethanol, and distilled water. Antigen retrieval was performed 1X antigen retriever solution (Sigma, pH 6.0). tissues were incubated with primary antibody in 5% donkey serum, 2% BSA, 0.5%TX-100 in TBS, followed by incubation of Alexa Fluor secondary antibody for 1.5 hr at room temperature, washed and counterstained with DAPI to visualize nuclei.

ROS Measurements

ROS detection reagent, 2′,7′-dichlorofluorescein (DCF) (Invitrogen, C6827) was used to determine intracellular ROS levels. DCF was reconstituted using anhydrous dimethylsulfoxide (DMSO, Sigma) with optimal working concentration at 10 μM in DMEM/2% serum. Media with DCF were added to cells in 96 wells and incubated for 1 hr in the dark at a 37 ºC, 5% CO2 incubator. After incubation, media with DCF was removed and cells were washed twice with PBS. DCF fluorescence intensity relative to background fluorescence in wells that do not contain cells was determined using a fluorescent microplate reader using excitation wavelength at 495nm and emission wavelength at 520nm. The results were normalized to cells/well. Data are displayed as Mean H2DCFDA fluorescence intensity ± SEM.

***In vitro* and *in vivo* cell proliferation**

Tumor cell proliferation in vitro was performed using the Premix WST-1 (Takara, Cat. MK400). Cells were seeded in 96 wells, flat bottom wells, in a 100 μl/well culture medium for the indicated time points; 10 μl/well Premix WST-1 was added to each well for 1 hr at tissue culture incubator. WST-1 is tetrazolium salt that is cleaved to formazan dye by succinate-tetrazolium reductase in viable cells, and measured at wavelength 420~480 nm. Tumor cell proliferation in vivo was measured by immunostaining of tumor sections using anti-Ki67 antibody (Abcam, AB15580). Two to three sections per tumor was stained, and 5 random fields per section were taken by using Zeiss fluorescence microscopy for counting Ki67 positive cells. Data are displayed as mean Ki67 positive cells ± SEM.

Transwell Matrigel Invasion

Cell invasion assays were performed in Corning Biocoat Matrigel Invasion chamber according to the manufacturer's instructions. Cell monolayers were made into single cell and plated at 1X105 cells/0.5 ml DME, 0.5% FBS per well into the upper compartment of the Boyden chamber for 18 hrs. Media with 10% serum were used in the lower chamber. Cells on top the filters were removed by cotton swabs, and cells penetrating the filters were stained with 0.5% crystal violet/1% formalin/20% methanol. Invaded cells were photographed and quantified by imageJ. The average number of invaded cells bound per microscopic field over five fields per assay was measured in triplicate experiments. Statistical significance was determined by one-tailed t-test.

Immunohistochemistry

Formalin-fixed/paraffin embedded tissues were sectioned at 5µm, deparaffinized in xylene, and rehydrated in a series of decreasing ethanol:H2O solutions. Antigens were retrieved by steam heat for 30 minutes in a 0.01 M pH 6.0 trisodium citrate buffer. Endogenous peroxidase activity was quenched by incubation of slides in 3% H_2O_2 for 10 min. Tissues were blocked in Dako Serum-Free Protein Block and incubated with primary antibodies in Invitrogen antibody diluent with 1% BSA at room temperature for 1 hr at room temperature or overnight at 4 ºC. After washes, tissues were incubated with Dako Envision Labeled Polymer-HRP anti-mouse or anti-rabbit secondary antibodies at room temperature for 30 min, washed, and developed by DAB substrate (Cell Marque, Cat. 957D). Sections, were washed and counterstained with Harris Haematoxylin (Fisher Scientific), followed by dehydration and mounting and validation by an expert breast pathologist. Quantitation of HIF1α staining was done on 4x of 20x fields of view from 6 tumors from 3 mice each group. Expression levels were scored based on staining intensity and area of tumor cells using a weighted histoscore calculated from the sum of (1 x 3% weak staining) + (2 x 3 % moderate staining) + (3 x 3 % strong staining) (2).

Vessel Perfusion

Tumor vessel perfusion assay was analyzed in three control and three mice bearing two mammary tumors on bilateral sites resulting in 6 tumors per group. Mice were injected intravenously with 50 µg DyLight® 594 labeled tomato lectin (Vector Laboratories, Cat# DL-1177) in saline. 10 minutes later, mice were euthanized and perfused with 1% PFA. Six mammary tumors were harvested from either control or GPx2 KD

tumor bearing mice, and frozen in optimum cutting temperature compound. Frozen sections were stained with anti-endomucin antibody (Santa cruz, sc-65495) that labels all vessels (functional and non-functional). Secondary antibody (Alexa Fluor 488 donkey anti-rat) was used to visualize endomucin labeled vessels. Perfusion ratio was calculated by dividing lectin-positive vessels by endomucin positive vessels using imageJ. Unpaired t-test was used to determine significance; $p<0.05$.

Vessel Maturation

Vessel maturation was analyzed by co-immunostaining of 6 control and 6 GPx2 KD mammary tumors with anti-endomucin antibody which labels vessels together withanti-laminin which stains basement membranes, or with anti-PDGFRβ which stains pericytes. Basement membrane or pericyte deficient vessels (immature) were mostly labeled with endomucin whereas mature vessels were labeled with endomucin and laminin. The maturation fraction of tumor vessels was quantified by ImageJ, and the maturation ratio was calculated by dividing laminin or positive vessels by entire endomucin labeled vessels.

Oxygen consumption in vitro

Cells were suspended in normal growth medium at a concentration of 1 $X10^4$ cells/ml, and 100 µl of cells was seeded on Seahorse 96-well plates 48 hrs prior to performing the assay. Oxygen consumption rate (OCR) assay was carried out in a XF96 Seahorse Analyzer (Seahorse Bioscience, Billerica, MA, USA). On the day of the assay, medium was replaced with prewarmed (37 °C) 180 µl Seahorse medium (Seahorse XF medium with 2 mM glutamine, 10 mM glucose, 1 mM pyruvate) and the plate was incubated for 1 hour in a 37 °C non-CO2 incubator. The mitostress test was performed with 1 µM oligomycin, 1 µM FCCP, and 0.5 µM rotenone/antimycin. The final OCR values were normalized to cells/well using the CyQUANT Cell Proliferation Assay Kit (Invitrogen, C7026).

Oxygen consumption in vivo

Tissue bioenergetics was determined using an XFe24 Seahorse respirometer. Briefly, tumor tissue was collected rapidly after sacrifice, cut into small pieces (8-12 mg) and quickly transferred to individual wells of an XFe24 plate. Individual pieces were stabilized from excessive movement by islet capture screens (Seahorse Bioscience), and 650µL Krebs-Henseleit buffer (KHB) (111 mM NaCl, 4.7 mM KCl, 2 mM MgSO4, 1.2 mM Na2HPO4, 0.5 mM carnitine, 2.5 mM glucose and 10 mM sodium pyruvate) were added to each well. Digitonin

(0.5 µg/mL, Sigma, D6628) was added to enhance plasma membrane permeability. Basal oxygen consumption rates (OCR) were determined at 37°C according to the following plan: Basal readings recorded every 2 min for 5 readings, followed by exposure to digitonin. Subsequent readings were recorded after 2 min mixing and 2 min rest. Basal OCR values were normalized to individual tissue weights.

Glycolysis stress test *in vitro*

Cells were suspended in normal growth medium at a concentration of 1 x10^4 cells/ml, and 100 µl of cells was seeded on Seahorse 96-well plates 48 hrs prior to performing the assay. Glycolysis stress assay was carried out in a XF96 Seahorse Analyzer (Seahorse Bioscience, Billerica, MA, USA). On the day of the assay, medium was replaced with prewarmed (37 °C) 180 µl of XF Base Medium (Agilent Technologies, 103335) supplemented with 2 mM glutamine and incubated for 1 hour at 37 °C in a non-CO_2 incubator. Baseline ECAR measurements were recorded 4 times (mix: 3 min; wait: 2 min; measure: 3 min), followed by sequential injection of 20 mM glucose, 1 µM oligomycin, and 50 mM 2-DG with 4 readings (mix: 3 min; wait: 2 min; measure: 3 min) after each injection. The ECAR values were normalized to cells/well using the CyQUANT Cell Proliferation Assay Kit (Invitrogen, C7026).

Cell isolation of mammary tumor cells for single cell RNA sequencing

PyMT1 control and PyMT1/GPx2 KD tumor (one tumor each) generated by injection of GFP-labelled tumor cell lines, was excised and mechanically chopped with scalpels, incubated in DMEM/F12 with 5% FBS, 5 µg/ml insulin, 2 mg/ml collagenase, and 150 µg/mL DNase at 37 °C for 45 min. Cell clamps were washed and dissociated with 2 ml of 0.05% trypsin/EDTA at 37 0C for 10 min and trypsinization stopped with 10ml PBS-2%FBS-EDTA. The samples were filtered through a 70 µm cell strainer, pelleted at 1200 rpm at 4 0C for 5 min, and resuspended in 1 ml of 1x red blood cell (RBC) lysis buffer and 10ml PBS-2%FBS-EDTA was added to stop lysis. Cells pellets were resuspended in 1ml PBS-2%FBS-EDTA and viability-controlled by trypan blue dye exclusion .GFP-labelled cells from PyMT1 control and PyMT1/GPx2 KD tumor were sorted using a FACSAria II (BD Biosciences).

Library Preparation and Single Cell RNA Sequencing

Library preparation was performed by Dr. David Reynolds at the AECOM genomic core facility. GFP-sorted single cell suspensions from one control PyMT1 tumor and one PyMT1/GPx2 KD tumor containing at

least 80% viable cells were processed to capture at least ~10,000 viable cells per sample. The libraries were prepared (individual lanes on the 10X Chromium) using the 10X Single Cell 3' v3 kit using ~10,000 cells per lane on the 10X Chromium microfluidics device (10X Genomics, Pleasanton, CA). The qualified libraries were sequenced on Illumina Hiseq 6000 platform (Novogene Corp, Sacramento, CA), with a standard paired-end 150bp (PE150) at each end.

The original data obtained from Illumina® HiSeq platform (Novogene Corp, Sacramento, CA), were transformed to sequenced reads by base calling. Raw data obtained as FASTQ files which contain sequenced reads were subjected to quality control using OpenGene's fastp v0.19.4 preprocessor (3). Cellranger 3.0.2. software was used to provide reads alignment and gene annotation to the mm10 mouse reference genome. The STAR aligner employed within cellranger 3.0.2 was used to perform alignment. Fragments Per Kilobase of transcript per Million mapped reads (FPKM) values were quantified using each cell-barcode in combination with unique molecular identifiers (UMIs) using the default cellranger. The quantified UMI counts were used to form an unfiltered gene-barcode matrix and exported for further downstream analysis.

Quality Control and Normalization

The unfiltered gene-barcode matrix for each sample was imported into R 3.6.1 and converted into SingleCellExperiment (SCE) objects. Any barcodes with a total UMI count greater than 200 were classified as cells. Seurat 3.1.1 was used for further quality control and preprocessing of the exported SCE objects. Cells with a minimum cut-off of 200 and a maximum cut-off of 4500 total RNA count were filtered. In addition, cells with a percentage of total reads that aligned to mitochondrial genome greater than 10% were removed (inferred as cells undergoing stress and cell death). After quality control, a total of 7621 cells from PyMT1 control tumor and 7768 cells from PyMT1/GPx2 KD tumor were used for normalization and downstream analyses. Cells passing QC were normalized using the SCTransform pipeline in Seurat 3.1.1.

Integration and Clustering

After normalization, the data obtained from PyMT1 control and PyMT1/GPx2 KD tumor underwent comprehensive integration using the integration pipeline in Seurat with the genes commonly retained between control and GPx2 KD tumor serving as integration anchors (4). Principal component analysis was performed to enable unsupervised clustering on the integrated data from PyMT1 control and PyMT1/GPx2 KD tumor, and

clustered cells were projected in two dimension using Uniform Manifold Approximation and Projection (UMAP) for cluster visualization (5). Louvain community detection algorithm was further applied to identify the cluster partitions, with a resolution parameter of 0.4 that was identified by building a clustering tree on the datasets to optimize the number of clusters.

Cluster Identification and Annotation

For cluster identification in the integrated dataset, we performed manually supervised-automated methods of cell-type identification. Canonical cell-type marker genes for basal (e.g, KRT5, KRT14) and luminal cells (e.g. KRT8, KRT18, CLDN) were annotated to identify which clusters belonged to which cell type. Seven epithelial clusters (cluster 0, 1, 2, 3, 4, 5, 6), and two non-epithelial clusters (cluster 7 and 8) were identified. Clusters 7 and 8 may be tumor associated stromal cells that co-sorted with GFP+ tumor cells. The number of cells identified per cluster in control versus GPx2 KD were as follows: cluster 0 (2391 vs 2472); cluster 1 (1781 vs 1796); cluster 2 (780 vs 1022); cluster 3 (983 vs 683); cluster 4 (819 vs 611); cluster 5 (715 vs 520); cluster 6 (205 vs 321); cluster 7 (128 vs 26); cluster 8 (119 vs 17). In total, 7621 control cells vs 7768 GPx2 KD cells were analyzed.

Cluster Comparison and Visualization

To compare cluster specific genes under two conditions, we used feature plot and violin plot functions to highlight expression patterns of marker genes of interest. Dot plots were used to visualize the average expression pattern of percentage and intensity of expression of genes of interest cells across all cell clusters.

Ingenuity Pathway Analysis

To investigate how GPx2 knockdown in PyMT1 tumor affects global gene expression, all genes regardless of subpopulations and differential expression status were ranked in decreasing order upon their average difference from the overall differential expression analysis without measuring clusters or cell state specific effect. For bulk analysis, clusters 7 and 8 were removed to get the overall differentially expressed genes. Genes with an adjusted p-value less than 0.05 were classified as differentially expressed. The dataset was uploaded to Ingenuity Pathways Analysis (IPA) www.ingenuity.com), for core analysis, and a p-value of 0.05 was used as a cut-off to determine statistically significant enrichment of a pathway or annotated gene

grouping present in the Ingenuity Knowledge base. A Z-score greater than +2 or less than -2 was used as a cut-off to predict a pathway activation or inhibition, respectively.

To investigate the upstream and downstream effects underlying functional activation or inhibition of OXPHOS or glycolysis pathways, overlay of the differentially expressed genes by GPx2 KD onto genes governing OXPHOS or glycolysis pathways in Ingenuity Knowledge Base allows IPA to determine functional activation or inhibition of oxygen consumption or glucose metabolism.

To investigate gene expression change by cluster comparison between PyMT1 control and PyMT1/GPx2 KD tumor, a joint variable of condition * cell-type was used to calculate cluster-type specific differential expression. Similarly, all differentially expressed genes belonging to specific cluster regardless of differential expression status were ranked in decreasing order based on their average difference. The data was then uploaded to IPA for core analysis.

TCGA Data Mining

The Tumor Cancer Genome Atlas (TCGA) breast cancer RNA-seq data was downloaded from the https://xenabrowser.net. Differences in GPx2 mRNA expression between tumor and matched normal breast tissue was established by paired t-test. Differences in expression of GPx2 mRNA between the four breast cancer subtypes (https://www.nature.com/articles/nature11412) were determined by the Kurksal Wallis test EdgeR (version 3.10) was used to generate a gene rank list of BC subtypes with low and high GPx2 mRNA expression. BC subtypes were divided into quartiles with quartile 1 representing 25% of tumors with the lowest GPx2 mRNA expression and quartile 4 with 25% of tumors expressing the highest levels of GPx2 mRNA.

Kaplan-Meier Analysis

Kaplan-Meier analysis comparing the expression levels of GPx1, GPx2, GPx3, GPx4 mRNA across all four breast cancer molecular subtypes with patient survival duration was performed using a breast cancer data set of 1809 patients using (http://kmplot.com/analysis/index.php?p=service&start=1).

Gene Set Enrichment Analysis

Gene set enrichment analysis (GSEA) was used to determine the gene sets enriched in breast cancer subtypes with low expression of GPx2 (https://www.ncbi.nlm.nih.gov/pubmed/16199517). Gene sets with a false discovery rate less than 0.15 were considered significant

Single Cell Sequencing data availability

The single cell RNA sequencing data reported in this manuscript are available at https://www.ncbi.nlm.nih.gov/geo with access number GSE152368. Coding analyses for single cell RNA sequencing data are available in Github with the URL accession link: https://github.com/Malindrie/Breast-cancer-scRNA-seq-analysis

Statistical Analysis

Results represent the Mean ± SEM (standard error of the Mean) for indicated experiments with 2-3 independent biological replicas. The statistical methods used are described in the figure legends. Most data were analyzed by the unpaired two- tailed Student's t test to compare two groups with significance set at the p value of < 0.05. Statistical analysis was performed using the Prism 8 (GraphPad) software.

SI References

1. X. Qian *et al.*, p21CIP1 mediates reciprocal switching between proliferation and invasion during metastasis. *Oncogene* **32**, 2292-2303 e2297 (2013).
2. E. C. Cheung *et al.*, Dynamic ROS Control by TIGAR Regulates the Initiation and Progression of Pancreatic Cancer. *Cancer Cell* **37**, 168-182 e164 (2020).
3. S. Chen, Y. Zhou, Y. Chen, J. Gu, fastp: an ultra-fast all-in-one FASTQ preprocessor. *Bioinformatics* **34**, i884-i890 (2018).
4. T. Stuart *et al.*, Comprehensive Integration of Single-Cell Data. *Cell* **177**, 1888-1902 e1821 (2019).
5. E. Becht *et al.*, Dimensionality reduction for visualizing single-cell data using UMAP. *Nat Biotechnol*, (2018).
